# Systematic review and meta-analysis of the effects of menopause hormone therapy on risk of Alzheimer’s disease and dementia

**DOI:** 10.3389/fnagi.2023.1260427

**Published:** 2023-10-23

**Authors:** Matilde Nerattini, Steven Jett, Caroline Andy, Caroline Carlton, Camila Zarate, Camila Boneu, Michael Battista, Silky Pahlajani, Susan Loeb-Zeitlin, Yelena Havryulik, Schantel Williams, Paul Christos, Matthew Fink, Roberta Diaz Brinton, Lisa Mosconi

**Affiliations:** ^1^Department of Neurology, Weill Cornell Medicine, New York, NY, United States; ^2^Department of Experimental and Clinical Biomedical Sciences, Nuclear Medicine Unit, University of Florence, Florence, Italy; ^3^Department of Population Health Sciences, Weill Cornell Medicine, New York, NY, United States; ^4^Department of Radiology, Weill Cornell Medicine, New York, NY, United States; ^5^Department of Obstetrics and Gynecology, Weill Cornell Medicine, New York, NY, United States; ^6^Department of Neurology and Pharmacology, University of Arizona, Tucson, AZ, United States

**Keywords:** meta-analysis, systematic review, menopause hormonal therapy, Alzheimer’s disease, dementia

## Abstract

**Introduction:**

Despite a large preclinical literature demonstrating neuroprotective effects of estrogen, use of menopausal hormone therapy (HT) for Alzheimer’s disease (AD) risk reduction has been controversial. Herein, we conducted a systematic review and meta-analysis of HT effects on AD and dementia risk.

**Methods:**

Our systematic search yielded 6 RCT reports (21,065 treated and 20,997 placebo participants) and 45 observational reports (768,866 patient cases and 5.5 million controls). We used fixed and random effect meta-analysis to derive pooled relative risk (RR) and 95% confidence intervals (C.I.) from these studies.

**Results:**

Randomized controlled trials conducted in postmenopausal women ages 65 and older show an increased risk of dementia with HT use compared with placebo [RR = 1.38, 95% C.I. 1.16–1.64, *p* < 0.001], driven by estrogen-plus-progestogen therapy (EPT) [RR = 1.64, 95% C.I. 1.20–2.25, *p* = 0.002] and no significant effects of estrogen-only therapy (ET) [RR = 1.19, 95% C.I. 0.92–1.54, *p* = 0.18]. Conversely, observational studies indicate a reduced risk of AD [RR = 0.78, 95% C.I. 0.64–0.95, *p* = 0.013] and all-cause dementia [RR = .81, 95% C.I. 0.70–0.94, *p* = 0.007] with HT use, with protective effects noted with ET [RR = 0.86, 95% C.I. 0.77–0.95, *p* = 0.002] but not with EPT [RR = 0.910, 95% C.I. 0.775–1.069, *p* = 0.251]. Stratified analysis of pooled estimates indicates a 32% reduced risk of dementia with midlife ET [RR = 0.685, 95% C.I. 0.513–0.915, *p* = 0.010] and non-significant reductions with midlife EPT [RR = 0.775, 95% C.I. 0.474–1.266, *p* = 0.309]. Late-life HT use was associated with increased risk, albeit not significant [EPT: RR = 1.323, 95% C.I. 0.979–1.789, *p* = 0.069; ET: RR = 1.066, 95% C.I. 0.996–1.140, *p* = 0.066].

**Discussion:**

These findings support renewed research interest in evaluating midlife estrogen therapy for AD risk reduction.

## Introduction

Neurodegenerative diseases associated with aging are a major public health concern, as the magnitude and proportion of populations aged 65 years and older continue to increase (Alzheimer’s Association, 2022). Alzheimer’s disease (AD) is the most common cause of dementia and the sixth leading cause of death in Western societies (Alzheimer’s Association, 2022). The number of persons living with AD dementia is projected to nearly triple by 2050 (Alzheimer’s Association, 2022) placing a considerable burden on already strained public health systems.

The female-based prevalence of AD is well documented with postmenopausal women accounting for over 60% of all those affected (Farrer et al., 1997; Altmann et al., 2014), an effect that is only partially explained by survival rates and longevity (Gao et al., 1998; Andersen et al., 1999; Lobo et al., 2000; Carter et al., 2012; Ferretti et al., 2018; Rahman et al., 2019). Thus, women emerge as a critical demographic for prevention action aimed at stemming the AD epidemic.

Mounting evidence from preclinical and translational studies identifies deprivation of estrogen’s neuroprotective effects following menopause, primarily 17β-estradiol, as a key biological underpinning of women’s AD risk (Brinton et al., 2015; Ferretti et al., 2018; Rahman et al., 2019). Consistent with these observations, the prodromal phase of AD, during which the disease is underway but symptoms are not yet manifest, can start as early as in midlife (Sperling et al., 2013), thus proximate to the menopause transition, or perimenopause. The characterization of the pre-symptomatic stage of AD is allowing development of primary and secondary prevention programs targeting at-risk individuals, thus before irreversible neuronal dysfunction and loss have occurred. Translational neuroimaging studies have shown that midlife perimenopausal and postmenopausal women at risk for AD exhibit increased biomarker indicators of AD risk, including higher Aβ deposition (Mosconi et al., 2017, 2018b, 2021; Rahman et al., 2020), brain glucose hypometabolism (Mosconi et al., 2017, 2018a,b, 2021; Rahman et al., 2020) and lower gray matter volume (GMV) in AD-vulnerable regions (Mosconi et al., 2017, 2018a,b, 2021; Kim et al., 2018; Rahman et al., 2020; Schelbaum et al., 2021) as compared to premenopausal women and/or age-controlled men. One study reported higher PET tau levels in postmenopausal women as compared to age-controlled men with comparable neocortical Aβ load (Buckley et al., 2022). Altered mitochondrial ATP production (Jett et al., 2022) and a higher burden of white matter hyperintensities (WMH) (Lohner et al., 2022) have also been observed with onset in perimenopause. Further, an earlier age at menopause, especially due to surgical menopause, has been associated with lower medial temporal lobe GMV (Zeydan et al., 2019), higher WMH load (Lohner et al., 2022), and greater neuropathological burden *in vivo* and *ex vivo* (Bove et al., 2014; Rahman et al., 2020; Coughlan et al., 2023) than spontaneous menopause. These observations, combined with preclinical evidence that perimenopause is a neuroendocrine transition associated with increased AD vulnerability (Brinton et al., 2015) and that estrogen therapy confers neuroprotective benefits (Brinton, 2008; Arevalo et al., 2015; Brinton et al., 2015), have spurred renewed interest in menopause hormone therapy (HT) for prevention of AD and dementia.

For decades, the association between HT use and dementia risk has been debated as findings from clinical studies have not been consistent. While multiple observational studies have indicated a protective association between HT use and reduced risk of AD or dementia (Henderson et al., 1994, 2005, 2007; Lerner et al., 1995; Mortel and Meyer, 1995; Paganini-Hill and Henderson, 1996; Tang et al., 1996; Kawas et al., 1997; Baldereschi et al., 1998; Harwood et al., 1999; Slooter et al., 1999; Waring et al., 1999; Zandi et al., 2002; Colucci et al., 2006; Rippon et al., 2006; Whitmer et al., 2011; Shao et al., 2012; Yoo et al., 2020; Kim et al., 2021; Depypere et al., 2022), there are currently no randomized controlled trials (RCTs) demonstrating AD reduction effects. The Women’s Health Initiative Memory Study (WHIMS), which remains the only RCT of HT effects on dementia incidence, reported a double increased risk of all-cause dementia with estrogen-plus-progestogen therapy (EPT), as well as a 50% increased risk with estrogen-only therapy (ET), which did not reach significance (Shumaker et al., 2003, 2004). The major limitation of the WHIMS is that the trials were conducted on postmenopausal women ages 65 and older, who were likely beyond the therapeutic window of estrogen efficacy (Henderson and Rocca, 2012; Rossouw et al., 2013; Manson et al., 2020). Results from ancillary studies, such as the WHIMS of younger women (WHIMS-Y), the Kronos Early Estrogen Prevention Cognitive and Affective Ancillary Study (KEEPS-cog) and the Early versus Late Intervention Trial with Estradiol-Cognitive Endpoints (ELITE-cog), indicate mostly neutral effects of HT on cognitive function among recently postmenopausal women (Shumaker et al., 2003, 2004; Gleason et al., 2015; Hodis et al., 2015).

The persistent debate surrounding the benefits and risks of HT in clinical studies may stem from the ‘one-size-fits-all’ approach of the WHIMS on the one hand, and the considerable heterogeneity of observational studies, in which multiple factors can influence HT efficacy and safety, on the other (Maki, 2005; Lethaby et al., 2008; Barrett-Connor and Laughlin, 2009; Maki and Sundermann, 2009; Marjoribanks et al., 2012; Weber et al., 2014; Conde et al., 2021; Stute et al., 2021). Meta-analyses are instrumental to reconcile these divergent findings, as they offer an integrative viewpoint on HT effects on AD risk by pooling results across different studies, while also taking into account variations in study design, populations, and treatment types, and systematically exploring possible sources of heterogeneity and bias.

Several meta-analyses examined HT effects on risk of AD or dementia (Yaffe et al., 1998; Hogervorst et al., 2000; LeBlanc et al., 2001; Farquhar et al., 2005, 2009; Marjoribanks et al., 2012, 2017; O’Brien et al., 2014; Song Y. J. et al., 2020; Wu et al., 2020; Zhang et al., 2021). However, many of these meta-analyses were completed nearly a decade ago, some even prior to the publication of WHIMS results, or at a time when only a limited number of prospective studies were available (Yaffe et al., 1998; Hogervorst et al., 2000; LeBlanc et al., 2001; Farquhar et al., 2005, 2009; Marjoribanks et al., 2012; O’Brien et al., 2014). Among these, those that focused solely on observational data generally reported protective effects of HT on AD or dementia risk, particularly with unopposed therapy, with risk reductions between 22% (O’Brien et al., 2014) and 46% (Hogervorst et al., 2000). In contrast, a series of Cochrane reviews, all of which limited to the WHIMS, reported an increase in dementia incidence (Farquhar et al., 2005, 2009; Marjoribanks et al., 2012, 2017). Recent meta-analyses have also produced contrasting results, likely attributable to differing selection criteria (Song Y. J. et al., 2020; Wu et al., 2020; Zhang et al., 2021). For instance, the study by Song Y. J. et al. (2020) that restricted analysis to observational data found a reduced risk of AD with HT use, whereas (Wu et al., 2020) incorporated data from the WHIMS, showing a mildly increased risk. The most recent study pooling estimates from existing meta-analyses and observational studies found an increased dementia risk with EPT but a decreased risk with ET (Zhang et al., 2021).

Overall, existing meta-analyses produced mixed results regarding the effect of HT on the risk of AD or dementia. Interpretation is complicated due to diverse selection criteria and methodological approaches across these studies. Additionally, most reports indicate substantial heterogeneity and variability across investigations, which was mostly associated with type of study (RCT vs. case–control vs. cohort), HT formulation (ET vs. EPT), and timing of initiation (midlife vs. late-life) (Yaffe et al., 1998; Hogervorst et al., 2000; LeBlanc et al., 2001; Farquhar et al., 2005, 2009; Marjoribanks et al., 2012; O’Brien et al., 2014; Song Y. J. et al., 2020; Wu et al., 2020). None of the existing meta-analyses stratified studies by formulation and initiation timing.

Accurate stratification has only become feasible with the recent publication of several large-scale observational studies. These studies report positive, negative and neutral effects of HT on dementia risk. Among positive findings, a case–control study examining health insurance claims from approximately 400,000 American women reported a protective effect of HT against AD, all-cause dementia, and other neurodegenerative diseases (NDDs) such as Parkinson’s disease, multiple sclerosis, and amyotrophic lateral sclerosis (Kim et al., 2021). Both ET and EPT users exhibited a reduced risk of AD and NDDs exceeding >50% compared to non-users, with greater risk reduction for long-term (>6 years) than short-term therapy (≤ 1 year) (Kim et al., 2021). A prospective study of 4.6 million women from Korea reported a 22% reduced risk of AD and 19% reduced risk of all-cause dementia with long-term HT of >5 years (Yoo et al., 2020). On the other hand, four Northern European studies reported an increased risk of dementia among HT users (Imtiaz et al., 2017a; Savolainen-Peltonen et al., 2019; Vinogradova et al., 2021; Pourhadi et al., 2023). A Finnish case–control study of 230,580 women reported a 10% increased risk of AD with HT started in midlife, with risk peaking after 6–10 years treatment for both opposed and unopposed therapy, but later inverting for ET and becoming non-significant for EPT after 10 years use (Imtiaz et al., 2017a). A population study of nearly 170,000 women, also from Finland, found a slightly increased risk of AD with ET use, and a significantly increased risk with EPT (Savolainen-Peltonen et al., 2019). Unlike the findings of Yoo et al. (2020) and Kim et al. (2021), the increased AD risk in women under 60 at HT initiation was associated with long-term exposure (>10 years) (Savolainen-Peltonen et al., 2019). In the UK, a case–control study of 615,917 women identified a 19% increased risk of AD with long-term EPT (>10 years), but found no significant effect of ET (Vinogradova et al., 2021). A nested case–control study of Danish national registries including 5,589 incident dementia and 55,890 age-matched controls found a 24% increased risk of all-cause dementia and 22% increased risk of AD with EPT started in midlife (Pourhadi et al., 2023). Dementia risk increased with longer duration of EPT use whereas AD risk reverted to non-significant after 12 years of use (Pourhadi et al., 2023). Finally, another case–control study of Danish national registries including 13,263 women found a non-significant 5% risk increase in all-cause dementia with overall HT use (Løkkegaard et al., 2022). None of these studies have been included in previous meta-analyses of HT effects on AD or dementia risk.

Herein, we conducted an updated systematic review and meta-analysis of scientific data linking HT to risk of AD and all-cause dementia, encompassing findings from 51 reports (45 observational studies and 6 RCT reports) up to the year 2023. To address some limitations of past meta-analyses, we employed multi-level meta-regression analysis to examine sources of heterogeneity, and considered from the outset the impact of variables such as study design, HT formulation, timing of initiation, and treatment duration. Further, we stratified studies by HT type and timing. We also discuss the biological plausibility of HT for AD prevention and extend our discussion to future considerations for clinical practice.

## Methods

### Search criteria

We conducted a systematic literature search in PubMed/MEDLINE, Web of Science, and Cochrane databases from 1975 through July 2023. Key words included (hormone replacement therapy, estrogen therapy, estrogen replacement therapy, postmenopausal hormone therapy) and (Alzheimer’s disease or Alzheimer or dementia). This search was augmented by a manual search of article bibliographies from topic reviews, meta-analyses and identified articles.

Screening of studies was conducted using a predesigned system by three independent authors (M.N., S.J., L.M.). Any discrepancies which arose during screening were resolved by the senior author (L.M.). The selection process involved 3 stages: (i) we removed all duplicate citations from the combined results from the searches using EndNote software (Thomson Reuters, New York, New York); (ii) we reviewed all citation titles and abstracts for relevance, and selected studies for full text review according to the inclusion criteria below; and (iii) we reviewed the full text of selected articles.

### Inclusion criteria

We selected only publications in the English language which met the following inclusion criteria: (1) the study was published in a peer-reviewed journal; (2) the cohort/study population was well defined; (3) outcome measures included AD or dementia incidence; (4) the study used a randomized placebo-controlled trial, case–control, cohort, or cross-sectional design (case reports, review papers, editorials, letters to the editor, personal communications, preclinical studies and *in vitro* research were excluded); (5) treatment was systemic estrogen with or without progestogen (studies of vaginal estradiol, tibolone, progesterone/progestin without estrogen, testosterone, and other preparations were excluded); (6) an estimate of association and at least one corresponding measure of statistical uncertainty such as *p* value, standard error, confidence interval, or data required for derivation of these estimates, were reported. These criteria were designed to identify high-quality studies and to ensure adequate data for meta-analysis.

### Data extraction

For each selected study, extracted data included year of publication, country, study design, number of participants, participants’ ages, number of AD or dementia vs. control cases, method for collecting data on hormone use, clinical endpoints (AD and/or dementia), HT characteristics (e.g., timing of use, duration of use, route of administration, formulation, or any available information), covariates, and summary estimates (odds ratio, OR, relative risk, RR, or hazard risk, HR) with 95% confidence intervals (C.I.). Nearly all studies (92%) included adjusted estimates. The fully adjusted models were primarily used for analysis.

### Statistical analysis

Statistical analyses were performed using R 4.2.2 statistical software (R Core Team). We *a priori* grouped study findings on the basis of study design (RCT vs. observational), HT categorization (any vs. never use, ET vs. EPT), and selected study characteristics included in the tables. For all examinations, we focused on systemic HT, including oral and transdermal preparations. Almost all studies reported hazard risk (HR), relative risk (RR) or odds ratio (OR) estimates. For those that did not, we calculated RR using data included in the papers (either in text, tables or supplemental materials) or reported estimates provided by previous meta-analyses. OR and HR point estimates and 95% C.I.s were converted to RR for each study. For the studies in which the outcome of AD or dementia was relatively rare (prevalence <15%) the RR was approximated as the reported HR or OR estimate (VanderWeele, 2020). For the subset of studies with greater prevalence, the RR was approximated using the following equation:


$$RR\approx \frac{1-0.5\sqrt{HR}}{1-0.5\sqrt{\frac{1}{HR}}}\;or\;\sqrt{OR}$$

Where the RR is approximately equal to one minus half the square root of the HR, divided by one minus half the square root of the inverse of the HR. For the OR, the RR can be directly approximated by the square root of the OR (VanderWeele, 2020). RR measures were log-transformed prior to performing the meta-analysis.

### Meta-analysis

Meta-analysis was performed for subgroups of at least four studies reporting comparable outcomes and exposure groupings (Mikolajewicz and Komarova, 2019; Higgins jpt and Li, 2022). For all meta-analyses, study-specific RR effect estimates were used to calculate pooled effect estimates and 95% C.I.s. These pooled estimates were calculated using a fixed or random-effects model which uses a weighting scheme that incorporates study sample size and within-and between-study variance (Jackson et al., 2010). We evaluated the presence of heterogeneity using Cochran’s Q, I^2^ and tau^2^ statistics (Higgins and Thompson, 2002). Random effects model estimates are presented for subgroups with significant study heterogeneity, whereas fixed effects model estimates are used for subgroups without significant heterogeneity. Unlike a fixed-effect model that assumes all studies share the same true underlying risk ratio, the random-effects model recognizes and accounts for the variability among different patient populations in the collected studies (Hedges and Olkin, 1985; DerSimonian and Laird, 1986). As a result, it allows estimating a mean risk ratio within a heterogeneous cohort, as appropriate. To further identify and minimize possible sources of heterogeneity, we (a) evaluated different exposures and outcomes in a multi-level meta-regression analysis, and (b) conducted a sensitivity analysis by examining factors such as study design, duration of therapy, HT formulation, and timing of use (see below).

Studies in the main analysis were stratified according to study design. RCTs included only postmenopausal women aged 65 and older who received oral conjugated equine estrogens (CEEs) with or without medroxyprogesterone acetate (MPA), with dementia as the primary outcome (Shumaker et al., 2003, 2004; Manson et al., 2013; Espeland et al., 2015). Consequently, RCTs were examined separately from observational studies to minimize heterogeneity.

For observational studies, our primary exposure was overall HT, characterized as use of any type of systemic HT vs. lack of use. Studies that only reported estimates for ET or EPT were included in the sensitivity analysis, below. For studies that presented multiple effect estimates, one estimate per study was included, as appropriate (Mikolajewicz and Komarova, 2019; Higgins jpt and Li, 2022). We performed separate meta-analyses to calculate pooled risk estimates for developing AD, and for risk of ‘AD plus dementia’ (all-cause dementia) in HT users compared with non-users. Only studies that reported specific AD risk estimates were included in analysis of AD risk. For analysis of all-cause dementia risk, estimates for AD were chosen as the primary outcome when available. When estimates of AD risk were not available, estimates for dementia were used. For completeness, we also provide estimates for ‘dementia plus AD’ by selecting dementia as the primary outcome (as opposed to AD). Whenever possible, overall estimates for treatment timing and duration were chosen. In line with guidelines for selecting the most relevant intervention or estimate for meta-analyses (Mikolajewicz and Komarova, 2019; Higgins jpt and Li, 2022), in cases where an overall estimate for timing was unavailable, estimates for midlife HT use were selected instead of late-life use. Similarly, when overall treatment duration data were unavailable, mid-duration use lengths were chosen.

Finally, for comparison with previous studies which include multiple effect estimates for different exposure types (Wu et al., 2020), we selected estimates following the following logic: (i) overall HT estimates were used wherever possible; (ii) where these were unavailable, estimates were provided for both ET and EPT, or either arm, as available. We then applied Robust Variance Estimation (RVE) which accounts for intra-study dependent effect sizes to compute the pooled effect size (VanderWeele, 2020).

### Examination of heterogeneity

To further examine and account for heterogeneity in our dataset, we employed a multi-level meta-regression analysis incorporating potential modifier variables using the ‘metafor’ package in R. This allowed us to obtain pooled RRs and 95% C. I adjusted for relevant sources of heterogeneity. Many studies in our dataset reported multiple effect sizes by exposure, initiation timing, duration of use, and/or HT type. While our primary meta-analysis utilized an estimate selection approach based on specific clinical criteria, the multi-level approach facilitated inclusion of multiple estimates per study, incorporating hierarchical structuring to account for induced correlations of estimates which may arise from any given study. This allowed us to account for within-and between-study dependencies across those parameters. An unstructured variance–covariance matrix was used for random effects variances (Cheung, 2014). Covariates included in the model include age at initiation, HT type, duration of use, study type, as well as time period (before 1995, 1995–2010, after 2010), study size (<500, ≥500) and effect estimate type (OR/RR/HR). Changes to effect sizes due to confounders were assessed at *p* < 0.05.

### Sensitivity analysis

We conducted sub-analyses testing for effects of:

- *Study design*. We conducted separate meta-analyses of observational studies grouped by study design (case–control vs. cohort). Cross-sectional studies were excluded.
- *Duration of use*. Only those estimates for studies in which duration-based categorical breakdowns are provided were included. Duration of use was specified as long or short. Long was defined as the longest duration category, with duration of use ≥3 years. Short was defined as duration of use <3 years. One study providing estimates for >6 months use (vs. <6 months) (Waring et al., 1999) was not included in analysis of long duration as the interval was deemed not long enough to warrant inclusion. Three studies providing estimates for 0–5 years of use as the shortest interval (Paganini-Hill and Henderson, 1996; Kawas et al., 1997; Imtiaz et al., 2017a) were not included in analysis of short duration as the interval was deemed not short enough to warrant inclusion.
- *HT formulation*. Only those estimates for studies in which the exposure was specified to be ET or EPT were included.
- *Time at HT use*. Only those estimates for studies in which women began HT in midlife or late-life were included.
- *HT formulation by timing*. Only studies providing estimates for HT use in midlife or late-life by HT formulation were included.

### Examination of publication bias

For analyses including 10 or more studies, presence of possible publication bias was evaluated using Egger’s tests and funnel plots for subgroups without significant heterogeneity (Egger et al., 1997). For subgroups with significant heterogeneity, we used the Trim and Fill method by Duval and Tweedie, which incorporates a random-effect model to estimate the presence of publication bias, and then imputes missing studies to the meta-analysis to account for the bias and re-compute the pooled effect size (Duval and Tweedie, 2000).

## Results

### Literature search and characteristics of included studies

**Figure 1:**
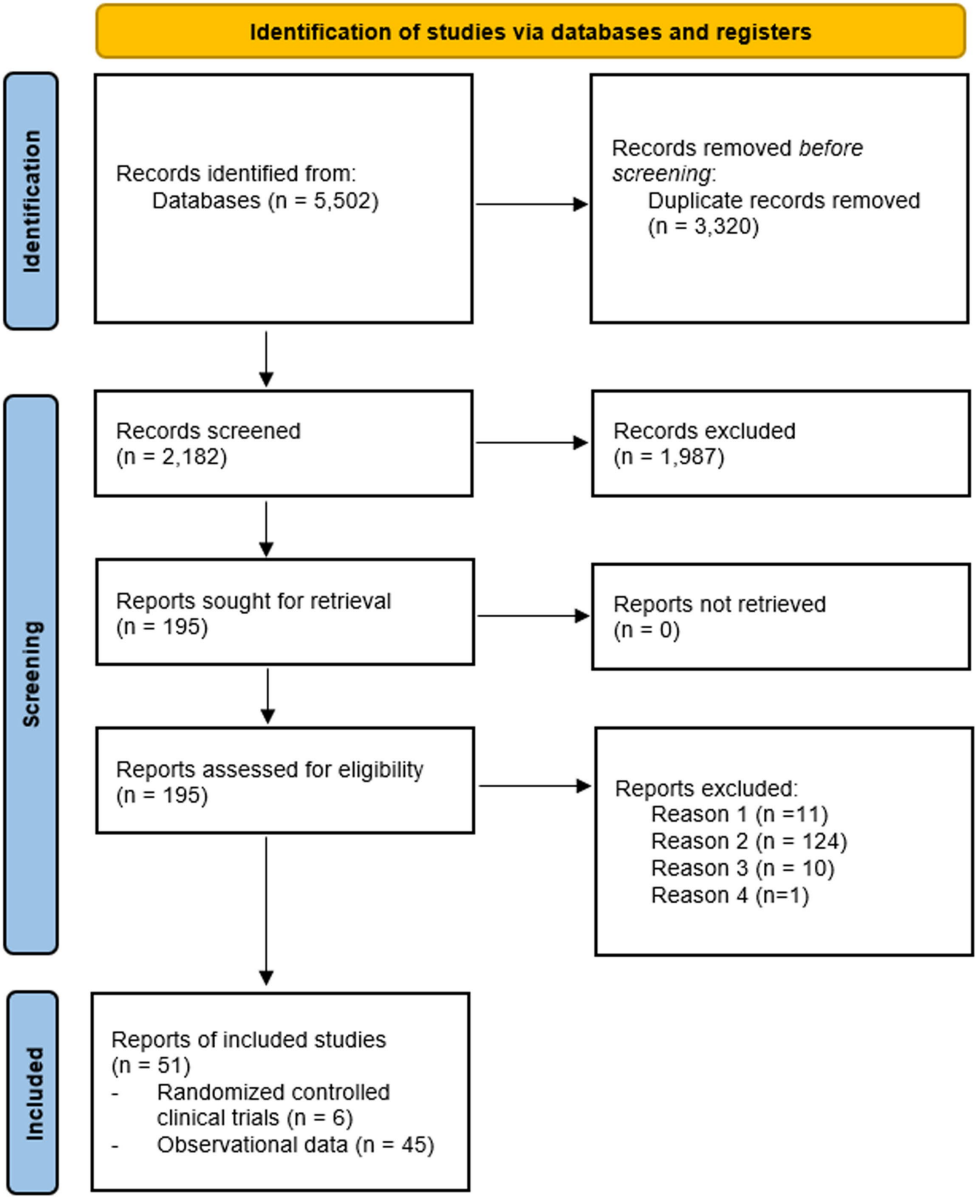
Flow chart Studies examining HT effects on risk of AD or dementia. Reason 1: meta-analysis. Reason 2: no assessment of AD or dementia risk. Reason 3: no OR/HR/RR estimates provided and/or no data to derive estimates. Reason 4: different estimates, e.g., standardized mortality rates.

Figure 1 presents a PRISMA flow chart of the systematic search and selection process. Through a comprehensive systematic search of PubMed/Medline, Web of Science, and Cochrane databases using established guidelines (Page et al., 2021), 5,502 papers were initially identified, of which 3,320 were found to be duplicates. From the remaining studies, 1,987 papers failed to meet the inclusion criteria and were excluded during title and/or abstract screening. The remaining 195 articles were selected for full-text scrutiny. Among these articles, a total of 51 eligible reports were pooled together for analysis (Figure 1).

Stratification of the studies:

- RCTs, *n* = 4 studies, including 6 reports, totaling 21,065 treated vs. 20,997 placebo participants. These include the two WHIMS trials, with separate reports for the two ET and EPT arms (Shumaker et al., 2003, 2004), and two long-term follow-ups of the WHIMS, also including two arms each (Manson et al., 2013; Espeland et al., 2015).
- Observational studies, *n* = 45 reports totaling 768,866 AD or dementia cases and 5.5 million controls. These include 24 case–control studies, 20 prospective cohort studies, and 1 cross-sectional study.

Key characteristics of included studies are found in Tables 1–5. By location, all RCTs were conducted in America. Among observational studies, 64% were conducted in America, 30% in Europe, and the rest in other countries. All studies collected information on HT use by self-report at the start of the study (e.g., interview or questionnaire), by review of electronic prescription databases, or by review of medical records.

**Table 1:** Randomized controlled clinical trials investigating effects of menopausal hormone therapy on dementia risk.

Reference	Design	Age, years	*N*	Outcome	Exposure distribution, %	Exposure*	Duration	N. cases	HR	95% C.I.
Shumaker et al. (2003)	4-year PC-RCT (WHIMS)	65–79	4,532	Dementia	EPT, 49%; placebo, 51%	Placebo		21	1.00	Ref.
						EPT		40	2.05	1.21–3.48
Shumaker et al. (2004)	5-year PC-RCT (WHIMS)	65–79	2,947	Dementia	ET, 50%; placebo 50%	Placebo		19	1.00	Ref.
						ET		28	1.49	0.83–2.66
	Pooled data with Shumaker et al. (2003)	65–79	7,479	Dementia	ET or EPT, 49%; placebo, 51%	Placebo		40	1.00	Ref.
						ET or EPT		68	1.76	1.19–2.60
Manson et al. (2013)	13-year post-intervention follow-up of WHIMS PC-RCTs	50–79	16,608	Dementia	EPT, 51%; placebo, 49%	Placebo		21	1.00	Ref.
						EPT		40	2.01	1.19–3.42
		50–79	10,739	Dementia	ET, 50%; placebo, 50%	Placebo		22	1.00	Ref.
						ET		33	1.47	0.85–2.52
Espeland et al. (2015)	18-year post-intervention follow-up of WHIMS PC-RCTs	65–80	7,233	Dementia	ET or EPT, 49%; placebo, 51%	Placebo	≤12-year follow up	142	1.00	Ref.
						ET	≤12-year follow up	146	1.02	0.73–1.43
						Placebo	≤12-year follow up	157	1.00	Ref.
						EPT	≤12-year follow up	175	1.32	0.98–1.77
						Placebo	≤18-year follow up	239	1.00	Ref.
						ET	≤18-year follow up	229	1.07	0.89–0.128
						Placebo	≤18-year follow up	300	1.00	Ref.
						EPT	≤18-year follow up	309	1.05	0.89–1.23

*In all studies, ET was oral CEE (0.625 mg/d); EPT was oral CEE (0.625 mg/d) and MPA (2.5 mg/d).

CEE, conjugated equine estrogens; EPT, estrogen-progestogen therapy; ET, estrogen therapy; HT, menopause hormone therapy; MPA, medroxyprogesterone acetate; PC-RCT, placebo-controlled randomized clinical trial; WHIMS, Women’s Health Initiative-Memory Study.

**Table 2:** Observational studies investigating effects of menopause hormone therapy on Alzheimer’s or dementia risk.

Reference	Country	Study design	Participants	Age, years*	Therapy	Alzheimer’s disease		Dementia	
						OR/HR/RR	95% CI	OR/HR/RR	95% CI
Heyman et al. (1984)	USA	Case–control	84	61	HT	2.38	0.7–7.8		
Amaducci et al. (1986)	Italy	Case–control	213	>65	HT	1.7	0.4–5.9		
Borenstein Graves et al. (1990)	USA	Case–control	120	65	HT	1.15	0.5–2.6		
Broe et al. (1990)	Australia	Case–control	170	79	HT	0.78	0.39–1.56		
Graves et al. (1990)	USA	Case–control	260	65	HT	1.15	0.50–2.64		
Paganini-Hill and Henderson (1994)	USA	Case–control	688	87	HT	0.67	0.38–1.17	0.69	0.40–1.20
Lerner et al. (1995)	USA	Case–control	157	>60	HT	0.41	0.30–0.90		
Mortel and Meyer (1995)	USA	Case–control	306	73	HT	0.6	0.3–1.2	0.48	0.20–1.20
Baldereschi et al. (1998)	Italy	Cohort	2,046	65–84	HT	0.28	0.08–0.98		
Harwood et al. (1999)	USA	Case–control	368	>65	HT	0.6	0.3–1.0		
Waring et al. (1999)	USA	Case–control	444	82	HT	0.47	0.20–1.0		
Seshadri et al. (2001)	UK	Case–control	283	66	HT	1.18	0.59–2.37		
Lindsay et al. (2002)	Canada	Cohort	2,079	73	HT	1.37	0.48–3.95		
Zandi et al. (2002)	USA	Cohort	1,866	75	HT	0.59	0.36–0.96		
Barnes et al. (2003)	USA	Cohort	577	76	HT	0.57	0.21–1.6		
Levine and Hewett (2003)	USA	Case–control	NR	70	HT	1.06	0.54–2.05	2.2	0.9–5.2
Colucci et al. (2006)	Italy	Case–control	405	75	HT	0.41	0.22–0.79		
Rippon et al. (2006)	USA	Case–control	1,498	68	HT	0.30	0.20–0.60		
Henderson et al. (2007)	USA	Cohort	7,100	NR	HT	0.40	0.20–0.85		
Ryan et al. (2009b)	France	Cohort	996	73	HT			1.55	0.6–3.97
Lau et al. (2010)	USA	Cross-sectional	4,087	77	HT	0.96	0.64–1.45	0.53	0.39–0.73
Whitmer et al. (2011)	USA	Cohort	3,447	49–76	HT			1.02	0.78–1.34
Shao et al. (2012)	USA	Cohort	1,768	75	HT	0.80	0.58–1.09		
Zucchella et al. (2012)	Italy	Case–control	551	77	HT	0.17	0.07–0.41		
Bove et al. (2014)	USA	Cohort	529	78	HT	0.91	0.74–1.13		
Imtiaz et al. (2017a)	Finland	Case–control	230,580	72	HT	1.10	1.06–1.12		
Imtiaz et al. (2017b)	Finland	Cohort	8,195	72	HT	1.10	0.83–1.40	1.08	0.88–1.3
Paganini-Hill et al. (2020)	USA	Cohort	424	69	HT			0.94	0.69–1.28
Song X. et al. (2020)	Singapore	Cohort	8,222	54	HT			0.61	0.46–0.80
Yoo et al. (2020)	Korea	Cohort	4,696,633	61	HT	0.77	0.70–0.86	0.81	0.78–0.84
Kim et al. (2021)	USA	Case–control	379,352	68	HT	0.43	0.41–0.46	0.41	0.4–0.43
Løkkegaard et al. (2022)	Denmark	Case–control	16,192	83	HT			1.05	0.93–1.19

*Mean age, unless stated otherwise.

HT, menopause hormone therapy; NR, not reported.

Most studies provided estimates for overall HT use. For studies reporting estimates for multiple outcomes, we selected the following: (Barnes et al., 2003), HT use for < 10 years; (Ryan et al., 2009b), past use; (Bove et al., 2014), estimates are reported for treatment initiation < 10 years vs. >10 years post-surgery for women who underwent oophorectomy; (Imtiaz et al., 2017a), midlife use; (Yoo et al., 2020), mid-duration of treatment (2–5 years); (Lau et al., 2010) reports on use of PIRx, potentially inappropriate medications with and without HT.

**Table 3:** Observational studies investigating effects of hormone therapy duration on Alzheimer’s or dementia risk.

Reference	Country	Study design	Participants	Age, years*	Duration (years or months)	Alzheimer’s disease		Dementia	
						OR/HR/RR	95% CI	OR/HR/RR	95% CI
Paganini-Hill and Henderson (1996)	USA	Cohort	1,488	88	>15 yrs	0.54	0.29–1.0		
Tang et al. (1996)	USA	Cohort	1,124	74	<1 yr	0.47	0.2–1.10		
					>1 yr. (mean 7 yrs)	0.13	0.02–0.92		
Kawas et al. (1997)	USA	Cohort	472	62	>10 yrs	0.50	0.17–1.47		
Waring et al. (1999)	USA	Case–control	444	82	<6 mo	0.85	0.44–1.62		
Seshadri et al. (2001)	UK	Case–control	283	66	12–25 mo	1.68	0.60–4.69		
					>60 mo	1.05	0.30–3.44		
Zandi et al. (2002)	USA	Cohort	1,866	75	<3 yrs	0.82	0.38–1.57		
					>10 yrs	0.41	0.17–0.86		
Barnes et al. (2003)	USA	Cohort	577	76	<10 yrs	0.57	0.21–1.6		
					>10 yrs	1.38	0.81–2.36		
Roberts et al. (2006)	USA	Case–control	486	84	0.5–3 yrs	1.22	0.47–2.20		
					>3 yrs	1.01	0.47–2.20		
Shao et al. (2012)	USA	Cohort	1,768	75	<3 yrs	0.68	0.38–1.22		
					>10 yrs	0.67	0.44–1.03		
Imtiaz et al. (2017a)	Finland	Case–control	230,580	72	0–5 yrs	1.10	1.06–1.14		
					>10 yrs	0.91	0.84–0.99		
Imtiaz et al. (2017b)	Finland	Cohort	8,195	72	1–3 yrs	1.00	0.72–1.50		
					>10 yrs	0.53	0.31–0.91		
Savolainen-Peltonen et al. (2019)	Finland	Case–control	169,468	<50 to ≥80	<3 yrs	0.89	0.69–1.15		
					>10 yrs	1.07	1.00–1.15		
Paganini-Hill et al. (2020)	USA	Cohort	424	69	<3 yrs			1.04	0.71–1.53
					≥15 yrs			0.85	0.69–1.64
Yoo et al. (2020)	Korea	Cohort	4,696,633	61	<2 yrs	0.86	0.81–0.92	0.86	0.84–0.88
					>5 yrs	0.78	0.71–0.87	0.87	0.84–0.92
Kim et al. (2021)	USA	Case–control	379,352	68	1–3 yrs	0.57	0.51–0.64	0.64	0.59–0.69
					>6 yrs	0.21	0.15–0.30	0.25	0.20–0.31
Vinogradova et al. (2021)	UK	Case–control	615,917	55–110	1–3 yrs	0.99	0.89–1.10	1.00	0.93–1.07
					>10 yrs	0.98	0.87–1.10	0.93	0.86–1.00
Pourhadi et al. (2023)	Denmark	Case–control	61,479	50–60	<1 year	1.04	0.83–1.29	1.21	1.09–1.35
					>12 yrs	1.24	0.85–1.81	1.74	1.45–2.1

*Mean age, unless stated otherwise.

Overall HT was selected as the primary exposure. When estimates for overall HT were not available, ET was selected, except for 1 study providing estimates for EPT only (Pourhadi et al., 2023). For studies providing multiple outcomes, estimates for midlife use were selected (Zandi et al., 2002; Shao et al., 2012; Savolainen-Peltonen et al., 2019; Pourhadi et al., 2023).

AD, Alzheimer’s disease; EPT, estrogen-progestogen therapy; ET, estrogen-only therapy; HT, menopausal hormone therapy.

**Table 4:** Observational studies investigating effects of hormone therapy formulation on Alzheimer’s disease or dementia risk.

Reference	Country	Study design	Participants	Age, years*	Therapy	Alzheimer’s disease		Dementia	
						OR/HR/RR	95% CI	OR/HR/RR	95% CI
Brenner et al. (1994)	USA	Case–control	227	78	ET	0.70	0.30–1.40		
Henderson et al. (1994)	USA	Case–control	235	76	ET	0.35	0.10–0.76		
Paganini-Hill and Henderson (1996)	USA	Cohort	1,488	88	ET	0.65	0.49–0.88		
Tang et al. (1996)	USA	Cohort	1,124	74	ET	0.40	0.22–0.85		
Kawas et al. (1997)	USA	Cohort	472	62	ET	0.46	0.21–1.00		
Slooter et al. (1999)	USA	Case–control	228	55	ET	0.34	0.12–0.94		
Seshadri et al. (2001)	UK	Case–control	283	66	ET	0.89	0.35–2.30		
					EPT	1.45	0.60–3.49		
Henderson et al. (2005)	USA	Case–control	971	50–99	ET	0.70	0.51–0.95		
Roberts et al. (2006)	USA	Case–control	486	84	ET	1.10	0.63–1.93		
Petitti et al. (2008)	USA	Cohort	2,906	79	ET	1.13	0.87–1.46		
					EPT	1.31	0.94–1.82		
Ryan et al. (2009a)	France	Cohort	3,130	>65	EPT			0.74	0.35–1.55
Shao et al. (2012)	USA	Cohort	1,768	75	ET	0.70	0.49–1.01		
					EPT	1.93	0.94–3.96		
Imtiaz et al. (2017a)	Finland	Case–control	230,580	72	ET	1.13	1.10–1.20		
					EPT	1.10	1.04–1.16		
Imtiaz et al. (2017b)	Finland	Cohort	8,195	72	ET	0.92	0.68–1.20	0.91	0.72–1.20
					EPT	1.10	0.87–1.50	1.10	0.89–1.40
Savolainen-Peltonen et al. (2019)	Finland	Case–control	169,468	<50 to ≥80	ET	1.06	1.01–1.12		
					EPT	1.14	1.09–1.19		
Paganini-Hill et al. (2020)	USA	Cohort	424	69	ET			1.02	0.73–1.42
Song X. et al. (2020)	Singapore	Cohort	8,222	54	ET			0.63	0.42–0.80
					EPT			0.59	0.40–0.86
Kim et al. (2021)	USA	Case–control	379,352	68	ET	0.34	0.23–0.50		
					EPT	0.25	0.21–0.30		
Vinogradova et al. (2021)	UK	Case–control	615,917	55–110	ET	0.89	0.79–1.01	0.92	0.85–1.00
					EPT	1.04	0.96–1.14	0.97	0.92–1.03
Pourhadi et al. (2023)	Denmark	Case–control	61,479	50–60	EPT	1.22	1.07–1.39	1.24	1.17–1.33

*Mean age, unless stated otherwise.

EPT, estrogen-progestogen therapy; ET, estrogen-only therapy; HT, menopausal hormone therapy.

Overall treatment duration was selected as the primary outcome whenever possible. For studies that reported multiple outcomes, we selected the following: (Petitti et al., 2008), midlife use; (Imtiaz et al., 2017a), medium duration of use (6–10 years); (Savolainen-Peltonen et al., 2019), midlife use; (Vinogradova et al., 2021), medium duration of use (3–5 years), (Pourhadi et al., 2023), midlife use.

**Table 5:** Observational studies investigating effects of timing of hormone therapy use on Alzheimer’s disease or dementia risk.

Reference	Country	Study design	Participants	Age, years*	Timing	Alzheimer’s disease		Dementia	
						OR/HR/RR	95% CI	OR/HR/RR	95% CI
Tang et al. (1996)	USA	Cohort	1,124	74	Midlife	0.40	0.22–0.85		
Kawas et al. (1997)	USA	Cohort	472	62	Midlife	0.46	0.21–1.00		
Waring et al. (1999)	USA	Case–control	444	82	Midlife	0.47	0.20–1.0		
Seshadri et al. (2001)	UK	Case–control	283	66	Late-life	1.18	0.59–2.37		
Zandi et al. (2002)	USA	Cohort	1,866	75	Midlife	0.33	0.15–0.65		
					Late-life	1.08	0.59–1.91		
Levine and Hewett (2003)	USA	Case–control	NR	70	Late-life	1.06	0.54–2.05	2.2	0.9–5.2
Henderson et al. (2005)	USA	Case–control	971	50–99	Midlife	0.35	0.19–0.66		
					Late-life	0.97	0.57–1.60		
Roberts et al. (2006)	USA	Case–control	486	84	Midlife	1.10	0.63–1.93		
Petitti et al. (2008)	USA	Cohort	2,906	79	Midlife	1.13	0.87–1.46		
Whitmer et al. (2011)	USA	Cohort	3,447	49–76	Midlife			0.74	0.58–0.94
					Late-life			1.48	1.10–1.98
Shao et al. (2012)	USA	Cohort	1,768	75	Midlife	0.70	0.49–0.99		
					Late-life	1.03	0.68–1.55		
Imtiaz et al. (2017a)	Finland	Case–control	230,580	72	Midlife	1.10	1.06–1.12		
Savolainen-Peltonen et al. (2019)	Finland	Case–control	169,468	<50 to ≥80	Midlife	1.06	1.01–1.12		
					Late-life	1.15	1.06–1.25		
Paganini-Hill et al. (2020)	USA	Cohort	424	69	Midlife			1.06	0.69–1.64
					Late-life			1.00	0.68–1.46
Kim et al. (2021)	USA	Case–control	379,352	68	Midlife	0.43	0.41–0.46	0.41	0.40–0.43
Pourhadi et al. (2023)	Denmark	Case–control	61,479	50–60	Midlife	1.22	1.07–1.39	1.24	1.17–1.33

*Mean age, unless stated otherwise.

HT, menopausal hormone therapy. Overall HT was selected as the primary exposure. When estimates for overall HT were not available, ET was selected (Tang et al., 1996), (Kawas et al., 1997), (Henderson et al., 2005), (Roberts et al., 2006), (Petitti et al., 2008), (Savolainen-Peltonen et al., 2019), except for Pourhadi et al. (2023) which only reports risk estimates for EPT.

### Clinical trials

The WHIMS remains the only RCT examining the effects of HT on incidence of all-cause dementia (Shumaker et al., 2003, 2004). Although AD was the primary outcome of interest, all-cause dementia became the default primary outcome because of the lack of sufficient numbers of AD cases at follow-up. Therefore, there are no RCTs that addressed effects of HT specifically on AD incidence. Further, given the time gap between typical menopause onset (age 51–52) and AD symptom onset (average age of 72 years in the U.S.), initiating a trial in early postmenopausal women would have required a 15–20-year follow-up. As this was impractical, the investigators chose to enrich the study by enrolling older women, ages 65 to 79 years, and without dementia at baseline (Shumaker et al., 2003, 2004). Women without a uterus were assigned to daily oral CEEs (oCEE, 0.625 mg) or placebo. Women with a uterus received oCEEs plus medroxyprogesterone acetate (MPA, 2.5 mg/day) in a continuous combined formulation, or placebo.

In the EPT arm, with a sample of 4,532 postmenopausal women, oCEE plus MPA treatment doubled the risk of all-cause dementia relative to placebo [HR = 2.05, 95% C.I. 1.21–3.48] (Shumaker et al., 2003). In the ET arm, with a sample of 2,947 postmenopausal women, oCEE use was associated with 49% increased risk of dementia relative to placebo, which did not reach significance [HR = 1.49, 95% C.I. 0.83–2.66] (Shumaker et al., 2004). Incidence rates for probable dementia in the ET trial were statistically similar to those in the EPT (45 vs. 22 cases per 10,000 person-years for oCEE plus MPA vs. matching placebo, respectively, *p* = 0.11). When data from the two trials were pooled, the overall HR for probable dementia was 1.76 [95% C.I. 1.19–2.60] (Shumaker et al., 2004). Of the dementia cases, 52% were classified as AD, 9% as vascular dementia (VaD), and 16% as mixed type, having features of both AD and VaD (Shumaker et al., 2004).

Our systematic review identified two follow-up studies to the WHIMS that reported dementia risk estimates several years after the trial was stopped (Manson et al., 2013; Espeland et al., 2015). In a 13-year post-intervention follow-up analysis, the HRs for probable dementia were still twice as high as placebo for the EPT arm [RR = 2.01; 95% C.I. 1.19–3.42], and 47% higher for the ET arm [RR = 1.47; 95% C.I. 0.85–2.52] (Manson et al., 2013). A subsequent study showed increased dementia risk after 12 years [HR = 1.18, 95% CI 0.95–1.48] but no significant effects after 18 years post-intervention [HR = 1.15, 95% C.I. 0.98–1.35] (Espeland et al., 2015).

**Figure 2:**
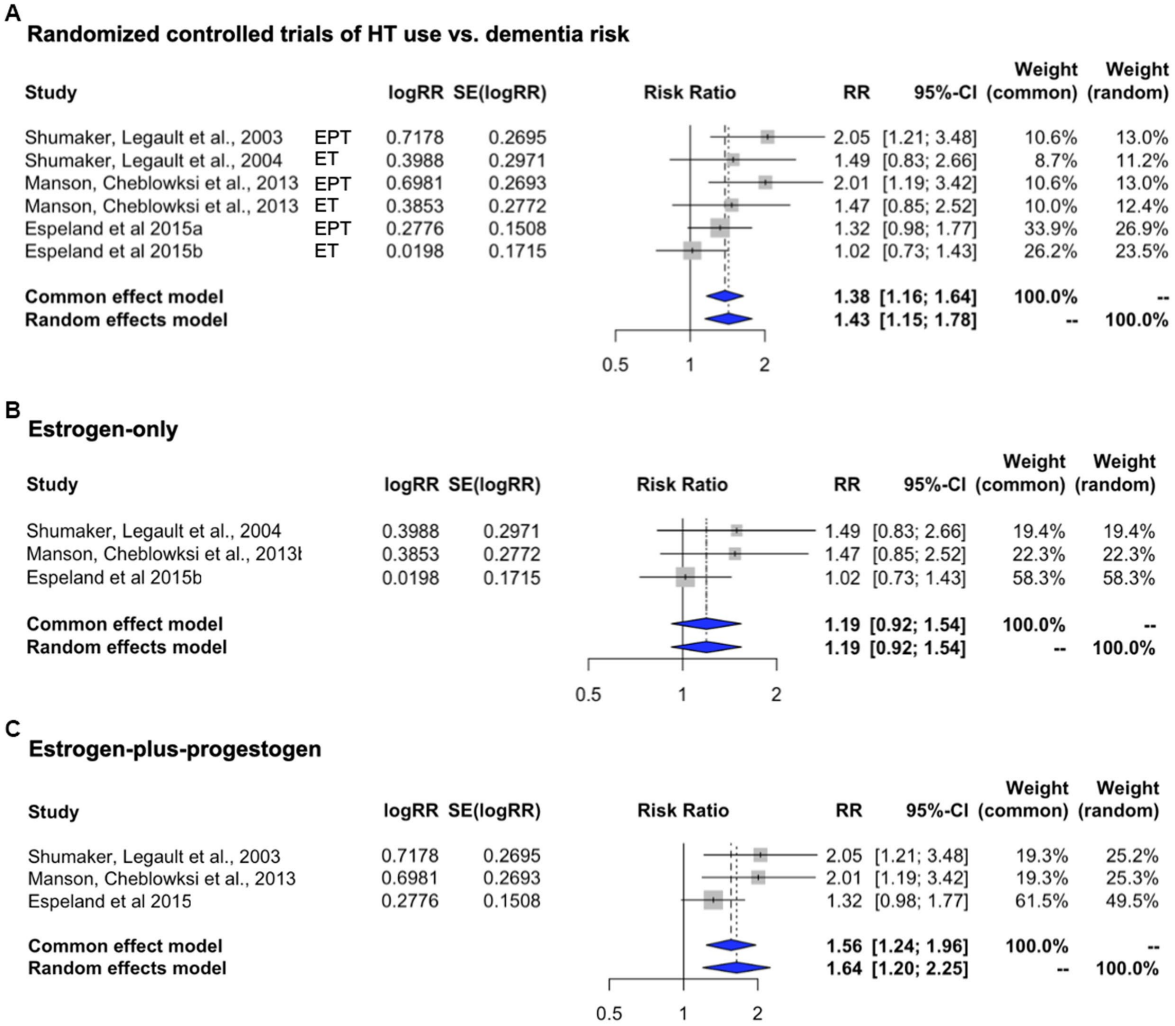
Meta-analysis of randomized, placebo-controlled trials of HT effects on dementia risk. Meta-analysis of randomized placebo-controlled trials investigating the risk of developing dementia with the use of systemic HT. Forest plots display individual and pooled estimates of the association between HT use **(A)** and dementia risk expressed as relative risk (RR) and 95% confidence intervals (C.I.). HT includes **(B)** estrogen-only (ET, oral conjugated equine estrogens, CEE) and **(C)** estrogen-plus-progestogen therapy (EPT, oral CEE and medroxyprogesterone acetate, MPA). Studies are ordered by year of publication.

Herein, we performed a meta-analysis to assess associations between HT and dementia risk by pooling data across the above reports (Table 1). As no significant heterogeneity was found to be occurring (*I*^2^ = 32.6%; tau^2^ = 0.024, *p* = 0.191), we interpreted the fixed effect model estimates. As shown in Figure 2A, overall HT use was associated with 38.1% increased risk of dementia compared with placebo [RR = 1.381, 95% C.I. 1.163–1.640; *p* < 0.001]. In this analysis, a mid-duration of treatment (up to 12 years) was selected for Espeland et al. (2015). Repeating analysis using the longest duration (up to 18 years) left results unchanged with a 28.2% increased dementia risk vs. placebo [RR = 1.282, 95% C.I. 1.116–1.472; *p* < 0.001].

In examination of HT type, only three studies were available per arm. Therefore, point estimates and C.I.s are presented for descriptive purposes, and we interpret the random effect model estimates. When compared to placebo, risk of dementia appeared to be higher for EPT [RR = 1.640, 95% C.I. 1.198–2.245] than with ET [RR = 1.191, 95% C.I. 0.922–1.540] (Figures 2B, C).

### Observational studies

Since the introduction of HT in the 1940s, several observational studies have been performed to determine its benefits and risks. The 1990’s and early 2000’s in particular showed great optimism regarding the benefits of estrogen therapy, encouraged by observational studies reporting a generally positive effect on AD incidence among women treated with HT compared with never-users (Henderson et al., 1994, 2007; Lerner et al., 1995; Mortel and Meyer, 1995; Baldereschi et al., 1998; Harwood et al., 1999; Slooter et al., 1999; Waring et al., 1999; Zandi et al., 2002; Colucci et al., 2006; Rippon et al., 2006), although negative reports were also present (Graves et al., 1990; Seshadri et al., 2001).

Notably, most of these studies were based on samples from the U.S. According to the Third National Health and Nutrition Examination Survey from 1992, American women who used HT at the time started therapy in response to menopausal symptoms, thus in midlife (Brett and Chong, 2001). Overall, 90% of those who used HT were younger than age 60 years (Wysowski et al., 1995). The most common type of HT used was ET, typically oCEEs (Wysowski et al., 1995). As a result, the hypothesis that HT protects against AD was based mainly on data reporting on ET, started in perimenopause or early postmenopause, and halted before age 60.

This time-of-initiation effect is exemplified in the prospective Cache County study, where HT initiated early after menopause was associated with over 40% reduced risk of AD compared to non-users, whereas HT initiated after age 60 doubled the risk (Zandi et al., 2002). In the prospective Rochester study, oophorectomy before menopause, but not after, was associated with an increased risk of AD (Rocca et al., 2007) – and ET initiated close to the time of surgery lowered this risk (Bove et al., 2014). These data suggested an apparent limited window of time during which estrogen exposure could reduce AD risk. As mentioned in the introduction, recent large-scale observational studies provide conflicting results of protective effects of both ET and EPT against AD (Yoo et al., 2020; Kim et al., 2021), while others show increased risk (Imtiaz et al., 2017b; Savolainen-Peltonen et al., 2019; Vinogradova et al., 2021; Løkkegaard et al., 2022). All these studies are integrated in this study.

Our systematic review yielded a total of 45 observational studies examining the relation between any type of HT and AD (n = 30), dementia (n = 6), or both (n = 9). According to our statistical analysis plan, these studies were stratified by exposure and outcomes.

In the main meta-analysis, we examined 32 studies reporting risk estimates for overall HT use. These included: (a) *all-cause dementia*: n = 32 studies reporting risk estimates for overall HT use vs. AD or dementia risk; (b) *AD-only*: n = 27 studies reporting risk estimates for overall HT use vs. AD risk (Table 2).

**Figure 3:**
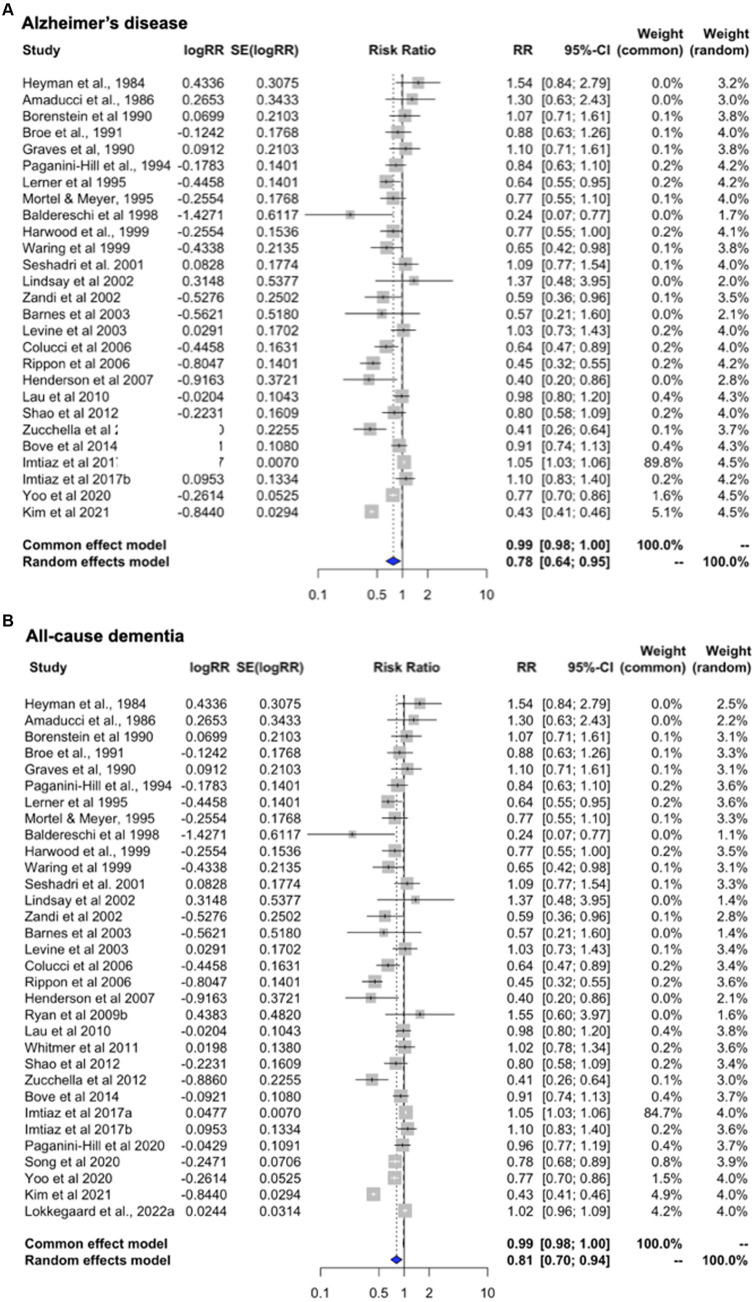
Meta-analysis of observational studies of overall HT use on AD or dementia risk Meta-analysis of observational studies examining the risk of developing AD or dementia with the use of systemic HT. Forest plots display individual and pooled estimates of the association between overall HT use and **(A)** AD and **(B)** AD or dementia risk expressed as relative risk (RR) and 95% confidence intervals (C.I.). Overall HT includes estrogen-only and estrogen-plus-progestogen formulations, oral or transdermal. Studies are ordered by year of publication. For all studies in panel **(A)** the outcome is AD incidence. In panel **(B)**, the outcome is AD plus dementia, by adding available estimates for all-cause dementia.

Heterogeneity was observed for studies of AD (*I*^2^ = 97.4%; tau^2^ = 0.226, *p* < 0.001) and all-cause dementia (*I*^2^ = 96.9%; tau^2^ = 0.147, *p* < 0.001). Therefore, we interpreted the random effects model results. Pooled estimates from random-effects meta-analysis indicated 22.2% reduced risk of AD [RR = 0.778, 95% C.I. 0.639–0.948, *p* = 0.013] (Figure 3A), and 18.9% reduced risk of all-cause dementia [RR = 0.811, 95% C.I. 0.70–0.94, *p* = 0.007] with overall HT use (Figure 3B). For completeness, we also generated estimates for ‘dementia plus AD’ by selecting dementia as the primary outcome instead of AD. Pooled estimates from random-effects meta-analysis indicated 20.1% reduced risk of all-cause dementia with overall HT use [RR = 0.807, 95% C.I. 0.684–0.952, *p* = 0.011], thus comparable to the analysis selecting AD as the primary outcome.

Finally, we generated estimates for the full unrestricted dataset (Supplementary Figure 1A). Where ET and EPT specific estimates are presented, the study is labeled accordingly in the plot. Pooled estimates from random-effects analysis indicate 16.3% reduced risk of developing AD or dementia in HT users vs. non-users [RR = 0.847, 95% C.I. 0.789–0.910, *p* < 0.001], consistent with results from the main meta-analysis. These estimates remained significant after applying RVE [RR = 0.814, 95% C.I. 0.737–0.892, *p* < 0.001].

### Examination of heterogeneity

As shown in Supplementary Table 1, the estimated risk ratio for AD with overall HT use, adjusting for the confounders, was 0.729 (0.465, 0.993), which suggests a protective effect of HT, consistent with the main meta-analysis (*p* < 0.001). HT type (*p* ≤ 0.021) and midlife use (*p* < 0.001) might be the sources of heterogeneity, as study design, duration of use, number of cases, publication year, and risk estimate had no moderating effects on the significant association between HT and AD incidence (*P’s* > 0.05, Supplementary Table 1). HT type and midlife use were associated with significant reductions in the RR (e.g., increased protective effect) as compared to the referent category of overall HT: the expected change in RR was greater for midlife use [RR change = −0.097.95% C.I. -0.132, −0.063, *p* < 0.001], followed by ET [RR change = −0.075; 95% CI -0.114, −0.036, *p* < 0.001] and EPT [RR change = −0.044, 95% C.I. -0.082, −0.007, *p* = 0.021]. Overall, this meta-regression results suggest that the primary drivers of heterogeneity in the risk of AD with HT are the age at which therapy is started, with midlife showing a protective effect, and the type of HT used, with ET showing a stronger protective effect than EPT. However, the remaining variance was still significant after accounting for these variables (*P* < 0.01). Possible sources of heterogeneity were further investigated in the sensitivity analysis, below.

## Sensitivity analysis

### Effects of study design

**Figure 4:**
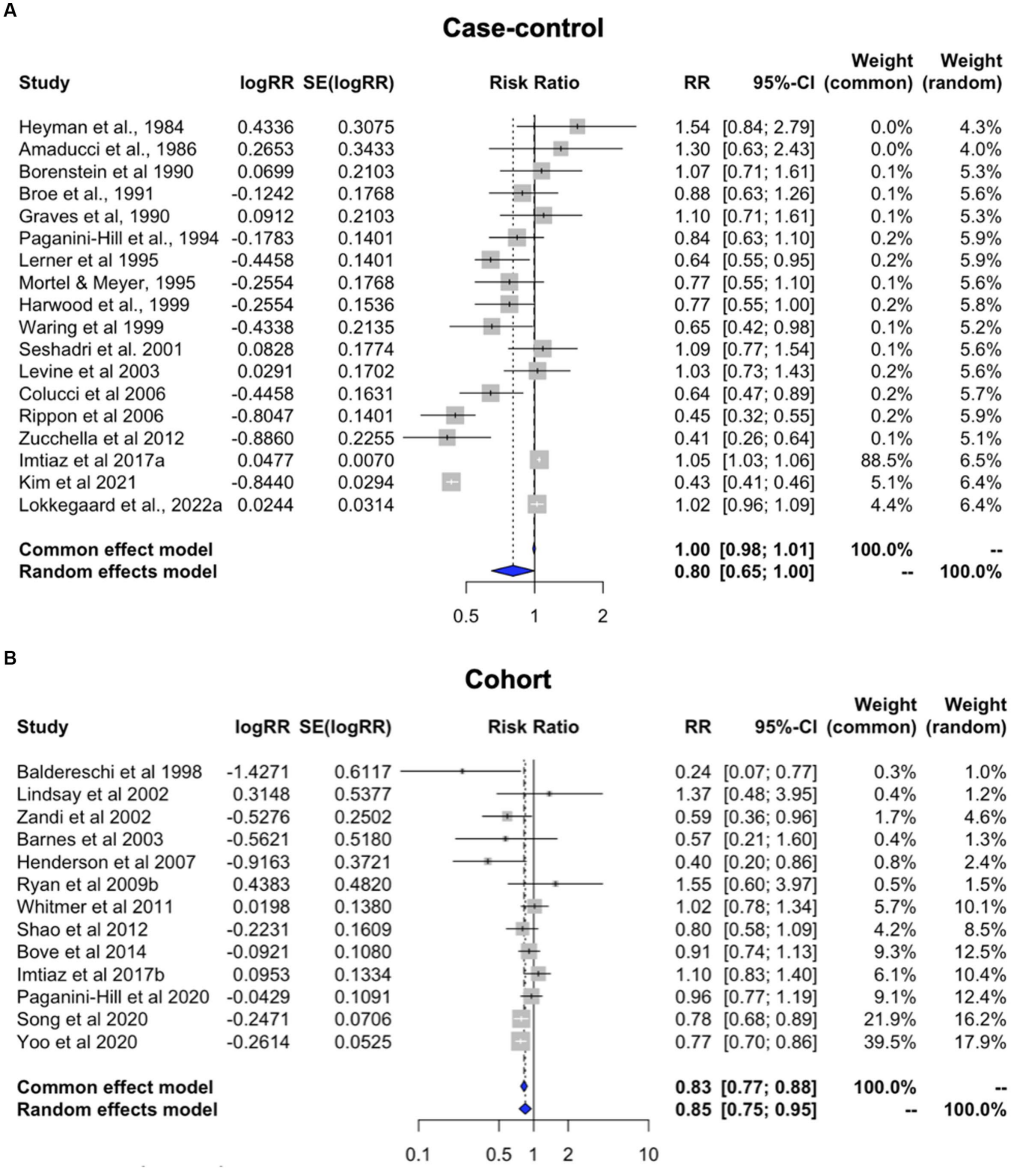
Meta-analysis of observational studies of overall HT use on AD or dementia risk by study design Meta-analysis of observational studies examining the risk of developing AD or dementia by study design. Forest plots display individual and pooled estimates of the association between use of HT in panel **(A)** case–control vs. **(B)** cohort studies and risk of AD or dementia expressed as relative risk (RR) and 95% confidence intervals (C.I.). Studies are ordered by year of publication.

We separately examined the case–control and cohort studies providing estimates for overall HT use which were included the main meta-analysis. Both subgroups exhibited significant heterogeneity (case–control: I^2^ = 98.2%, tau^2^ = 0.192, *p* < 0.001; cohort: I^2^ = 51.8%, tau^2^ = 0.018, *p* = 0.015). Pooled estimates from random effects meta-analysis indicate a 19.6% reduced risk of dementia for case–control studies [RR = 0.804; 95% C.I. 0.646–0.999, *p* = 0.050] (Figure 4A) and 15.4% reduced risk for cohort studies [RR = 0.846; 95% C.I. 0.750–0.954, *p =* 0.006] (Figure 4B).

### Effects of duration of HT use

This analysis includes studies providing estimates for a long duration of use (average > 10 years) and for a short duration of use (<3 years) in relation to AD or dementia risk (Table 3). All studies except two reported risk estimates for AD as the primary outcome.

**Figure 5:**
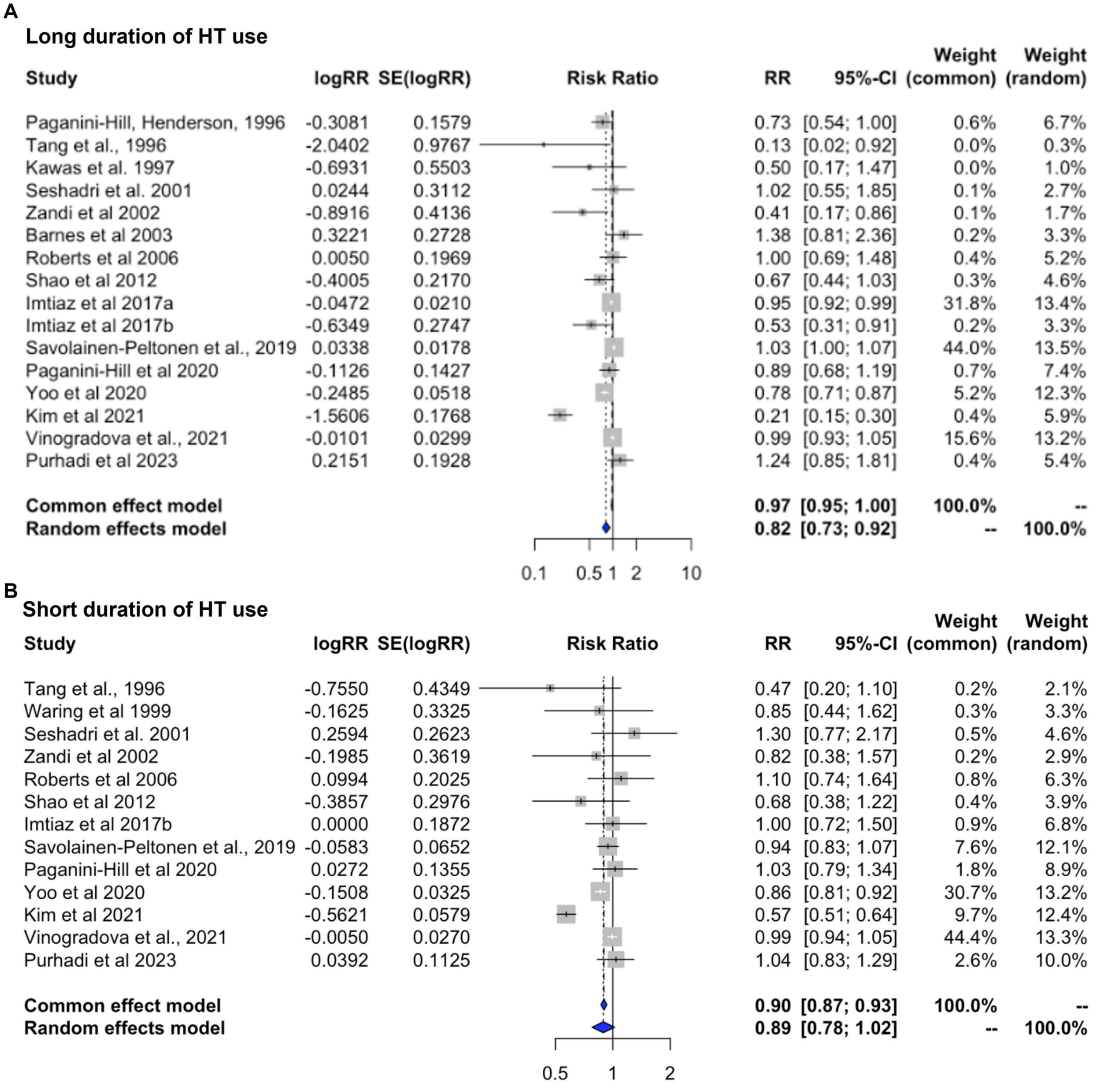
Meta-analysis of observational studies of short vs. long duration of HT use on AD or dementia risk Meta-analysis of observational studies examining the risk of developing AD or dementia by duration of HT use. Forest plots display individual and pooled estimates of the association between **(A)** long vs. **(B)** short duration of HT use and risk of AD or dementia expressed as relative risk (RR) and 95% confidence intervals (C.I.). Studies are ordered by year of publication.

Heterogeneity was found for studies reporting on long and short duration of HT use (I^2^ = 88.6%, tau^2^ = 0.024, and I^2^ = 86.3%, tau^2^ = 0.035, *p* < 0.001, respectively). Pooled estimates from random effects meta-analysis indicated 18.1% reduced risk of dementia with long duration of HT use [RR = 0.819; 95% C.I. 0.732–0.916, *p* < 0.001] (Figure 5A), and a non-significant 10.9% reduced risk with short duration of use [RR = 0.891; 95% C.I. 0.778–1.021, *p* = 0.097] (Figure 5B). All studies of long duration included estimates for >5 years HT use, except for Tang et al. (1996), which reported estimates for >1 year for an average treatment duration of 7 years, and (Roberts et al., 2006) reporting estimates for >3 years use (Table 3). As these studies were not weighted prominently in the meta-analysis (0.4 and 5.5%, respectively), removing them did not significantly impact results.

### Effects of HT formulation

**Figure 6:**
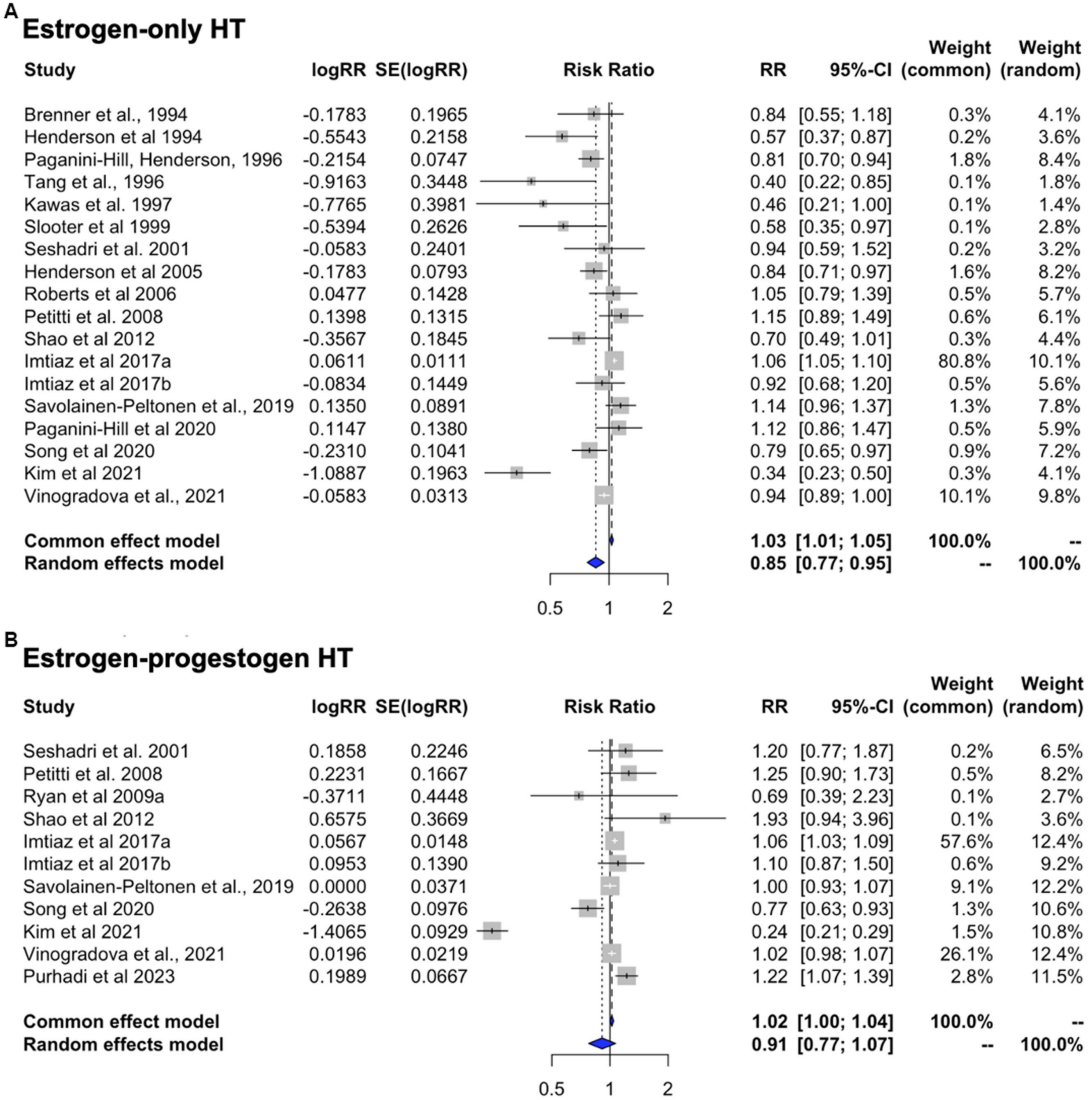
Meta-analysis of observational studies of estrogen-only vs. estrogen-plus-progesterone HT use on AD or dementia risk Meta-analysis of observational studies examining the risk of developing AD or dementia by HT formulation. Forest plots display individual and pooled estimates of the association between use of **(A)** estrogen-only vs. **(B)** estrogen-plus-progestogen therapy and risk of AD or dementia expressed as relative risk (RR) and 95% confidence intervals (C.I.). Studies are ordered by year of publication.

This analysis includes only those estimates for studies in which the exposure was specified to be ET or EPT, with all-cause dementia as the primary outcome (Table 4). Heterogeneity was observed for both ET (*I*^2^ = 83.8%; tau^2^ = 0.026, *p* < 0.001) and EPT (*I*^2^ = 96.2%; tau^2^ = 0.054, *p* < 0.001). Pooled estimates from random-effects meta-analysis indicated 14.5% reduced risk of dementia for ET [RR = 0.855, 95% C.I. 0.773–0.945, *p* = 0.002] (Figure 6A), and a non-significant 9.0% reduced risk for EPT [RR = 0.910, 95% C.I. 0.775–1.069, *p* = 0.251] (Figure 6B).

We then repeated analysis including only the studies reporting estimates for AD risk (Table 4). This yielded comparable results, with 17.7% reduced risk of developing AD for ET [RR = 0.823, 95% C.I. 0.734–0.922, *p* < 0.001], and a non-significant 9.9% reduced risk for EPT vs. non-use [RR = 0.901, 95% C.I. 0.753–1.078, *p* = 0.256].

### Effects of HT timing

**Figure 7:**
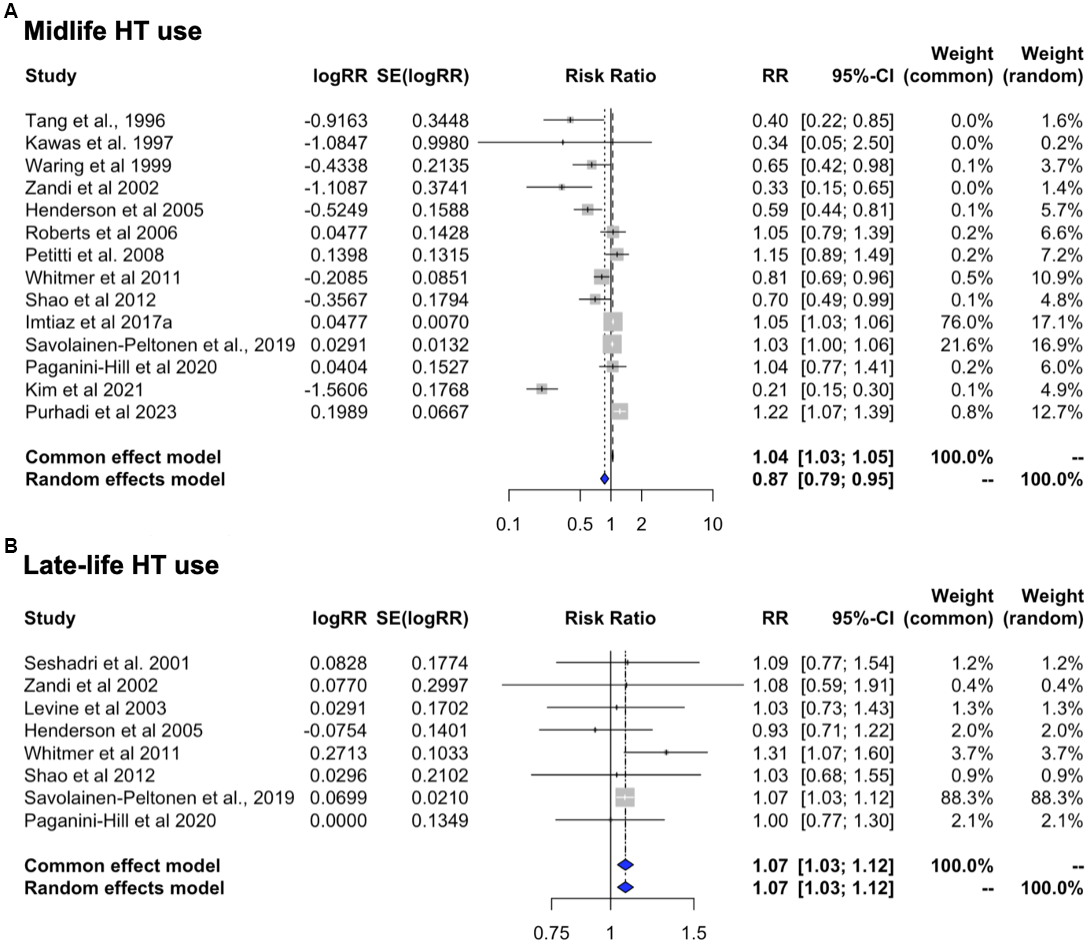
Meta-analysis of observational studies of midlife vs. late-life HT use on AD or dementia risk Meta-analysis of observational studies examining the risk of developing AD or dementia by timing of HT. Forest plots display individual and pooled estimates of the association between use of HT in **(A)** midlife vs. **(B)** late-life and risk of AD or dementia expressed as relative risk (RR) and 95% confidence intervals (C.I.). Studies are ordered by year of publication.

This analysis includes studies of HT use in midlife or late-life, with all-cause dementia as the primary outcome (Table 5). Heterogeneity was observed for studies of midlife use (I^2^ = 90.7%, tau^2^ = 0.013, *p* < 0.001) but not for late-life use (I^2^ = 0.0%, tau^2^ = 0.0, *p* = 0.632). Therefore, random effects are interpreted for midlife use and fixed effects for late-life use. Pooled estimates from meta-analysis indicated a 13.3% reduced risk of dementia with HT use in midlife [RR = 0.867; 95% C.I. 0.792–0.950, *p* = 0.002] (Figure 7A). In contrast, HT use in late-life was associated with 7.5% increased dementia risk [RR = 1.075; 95% C.I. 1.034–1.117, *p* < 0.001] (Figure 7B).

We then examined the studies that reported estimates for AD risk with any HT use in midlife or late-life. This showed a 15.9% risk reduction with midlife use [RR = 0.841, 95% C.I. 0.758–0.933, *p* = 0.001] and a 6.8% increase in AD risk with late-life use [RR = 1.068, 95% C.I. 1.027–1.112, *p* = 0.001].

### Effects of HT by formulation and initiation timing

In an effort to provide an integrative interpretation of the available data, taking into consideration both type of treatment and initiation timing, we performed a meta-analysis of the 22 studies offering stratification based on these parameters. These include 14 reports of midlife use (*n* = 8 ET; *n* = 6 EPT) and 8 reports of late-life use (*n* = 4 ET, *n* = 4 EPT). In the majority of studies (77%), AD was the primary outcome.

**Figure 8:**
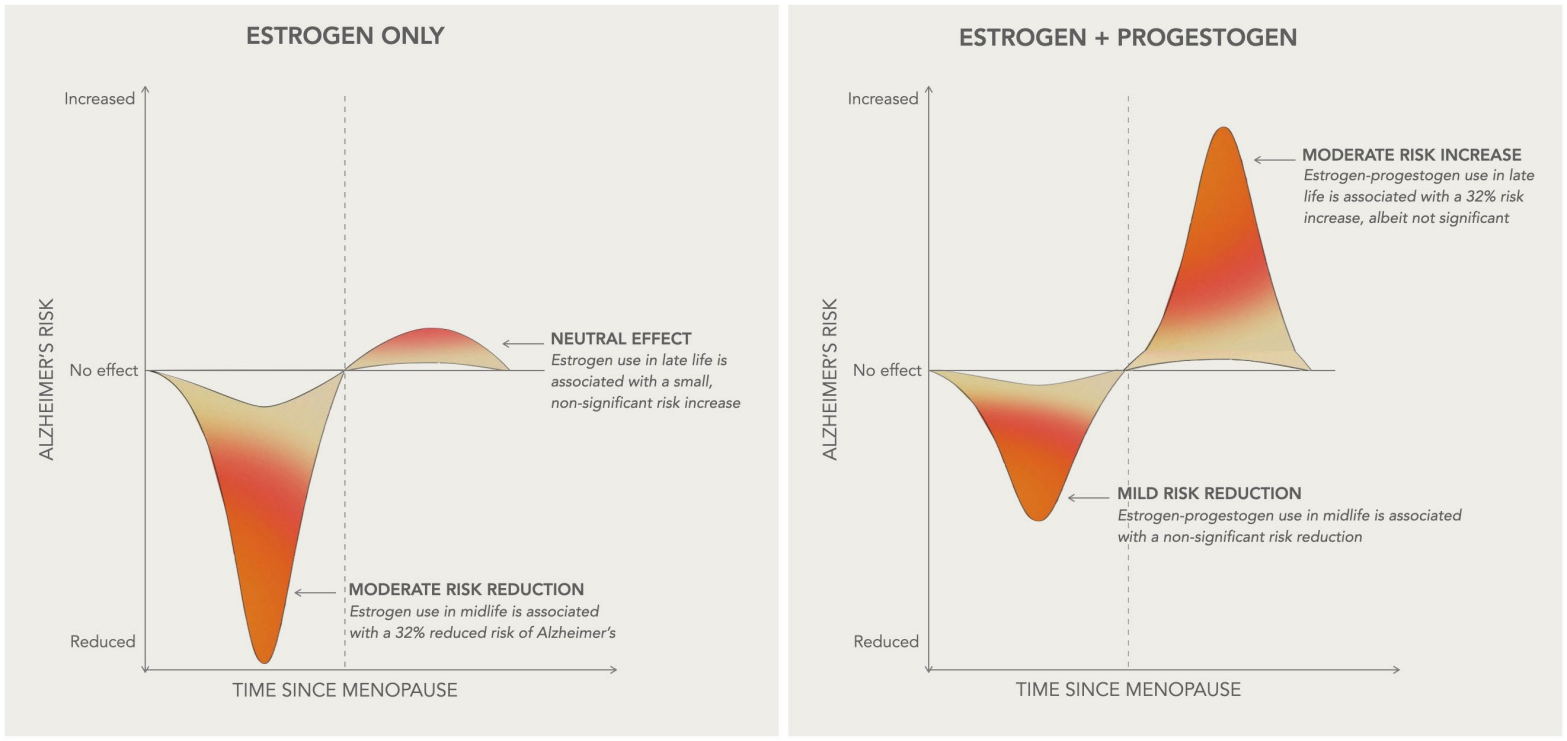
Summary of HT effects on AD risk Schematic overview of the main results of the study, illustrating how HT efficacy on AD risk varies based on initiation timing relative to menopause onset and HT formulation. In midlife, or more generally within 10 years of the final menstrual period, estrogen-only therapy is associated with a moderate decrease in AD risk, while estrogen-progestogen therapy is associated with a milder, non-significant risk reduction. In late-life, or more than 10 years after menopause, estrogen-only therapy presents neutral effects on AD risk, whereas estrogen-progestogen therapy is associated with a moderate risk increase, albeit non-significant.

In midlife, ET use was associated with 31.5% reduced risk of dementia as compared to non-use [RR = 0.685, 95% C.I. 0.513–0.915, *p* = 0.010] while EPT use was associated with 22.5% reduced risk, which did not reach significance [RR = 0.775, 95% C.I. 0.474–1.266, *p* = 0.309]. In late-life, both regimens showed trends toward an increased risk of dementia, estimated at 6.6% for ET [RR = 1.066, 95% C.I. 0.996–1.140, *p* = 0.066] and 32.3% for EPT [RR = 1.323, 95% C.I. 0.979–1.789, *p* = 0.069]. These effects are summarized in Figure 8.

### Publication Bias

Egger’s tests were not applicable to RCTs due to the limited number of reports. Qualitatively, funnel plots did not show clear asymmetry (Supplementary Figure 2). Duval and Tweedie Trim and Fill tests indicated no significant publication bias in the main analysis of observational studies (AD-only *p* = 0.303; all-cause dementia *p* = 0.214) or in subgroup analyses (*P*’s > 0.255; Supplementary Figure 2). Overall, these results suggest that the findings of this meta-analysis were unlikely to be impacted by severe publication bias.

## Discussion

The present meta-analysis, examining data from 51 reports encompassing 21,065 HT treated vs. 20,997 placebo participants and 768,866 AD or dementia cases vs. 5.5 million controls, indicates an association between HT use and risk of AD or dementia. This association varied depending on timing of initiation, and to a lesser extent, HT formulation. In midlife, ET use was associated with a 32% reduction in dementia risk, while EPT use showed a non-significant 23% risk reduction as compared to non-use. Conversely, late-life use of both formulations was associated with an increased dementia risk, more so with EPT than ET, although neither reached statistical significance. Additionally, observational data suggest increasing benefits with longer duration of HT use, while study design did not affect the outcomes.

Collectively, research thus far indicates a critical window for therapeutic benefit for HT, especially ET, within the context of the healthy cell bias of estrogen action (Brinton, 2008), as discussed below.

### Biological plausibility and the “healthy cell bias” hypothesis

The relationship between sex hormones and cognitive function has been under extensive investigation over the last three decades. There are almost innumerable biological reasons why estrogen, primarily 17β-estradiol, could be protective against memory loss and AD (Morrison et al., 2006; Brinton et al., 2015). Estradiol exerts multiple regulatory actions in the central nervous system mediated by direct effects on neurons and glial cells, including effects on neuronal morphology, number, and metabolic activity (McEwen, 1981; Behl, 2002; Arevalo et al., 2015). It supports neuronal plasticity through genomic and non-genomic actions (McEwen et al., 1997; Brinton et al., 2015; Lai et al., 2017), increases in spinogenesis and synaptogenesis (Hara et al., 2015), cell proliferation (Goodman et al., 1996), and gene expression (Woolley and McEwen, 1992; Brinton, 2008; Yin et al., 2015). Further, estradiol is considered a “master regulator” of brain bioenergetics, acting as a critical signaling molecule involved in glucose uptake and metabolism, mitochondrial respiration, and ATP generation (Rettberg et al., 2014; Brinton et al., 2015). As such, it plays a major role in regulating mitochondrial pathways, promoting aerobic glycolysis and the citric acid cycle (TCA) by increasing the activity of glycolytic and TCA enzymes (Rettberg et al., 2014), pyruvate dehydrogenase (PDH), and ATP synthase (Nilsen et al., 2007).

The transition to menopause is marked by radical changes in the production and activity of estradiol in body and brain (McEwen et al., 1997), resulting in a bioenergetic deficit characterized by downregulation of glucose transporter 3 (GLUT3), pyruvate dehydrogenase 1 (PDH1), and oxidative phosphorylation (Yao et al., 2011, 2012; Ding et al., 2013; Yin et al., 2015). Concomitant reductions in cerebral metabolic rates of glucose (CMRglc), as detected by positron emission tomography (PET), are apparent in clinical analyses of oophorectomized women as well as those undergoing spontaneous menopause (Mosconi et al., 2017, 2018a,b, 2021; Rahman et al., 2020). CMRglc decline is also found during the prodromal phase of AD (Mosconi, 2005) and can activate inflammatory processes involved in AD pathophysiology (Mishra and Brinton, 2018; Mishra et al., 2020; Wang et al., 2020a,b). Direct links between estrogen and AD pathology have also been reported, as estradiol promotes non-amyloidogenic processing by upregulating Aβ-degradation enzymes (Merlo and Sortino, 2012), increasing secretion of amyloid precursor protein (APP), and decreasing Aβ production (Xu et al., 1998; Manthey et al., 2001; Nord et al., 2010), and via its action on insulin degrading enzyme, a protease involved in Aβ degradation (Zhao et al., 2011). Further, estradiol reduces levels of both induced and naturally occurring hyperphosphorylated tau protein (Alvarez-de-la-Rosa et al., 2005; Liu et al., 2008). Estrogen withdrawal following oophorectomy increases tau hyperphosphorylation, inflammation, and Aβ-induced neurotoxicity in AD transgenic female animals (Li et al., 2000; Levin-Allerhand et al., 2002; Yue et al., 2005; Nilsen et al., 2006). Translational studies in humans also report higher Aβ deposition (Mosconi et al., 2017, 2018b, 2021; Rahman et al., 2020) and tau pathology (Buckley et al., 2022) in postmenopausal women, with oophorectomized patients exhibiting greater neuropathological burden as compared to those undergoing spontaneous menopause (Bove et al., 2014; Rahman et al., 2020; Coughlan et al., 2023). Collectively, these findings suggest a role for estrogenic preservation in AD prevention by means of HT interventions.

Preclinical discoveries led to the proposition of a “healthy cell bias of estrogen action” which posits that women may benefit from estrogen therapy when treated at the time of the menopause transition, and before neurological compromise (Brinton, 2008). The healthy cell bias hypothesis highlights effects of early versus later initiation, and may provide explanation for the mixed results and divergent outcomes of HT on AD risk, as there may be a threshold up to which healthy neurons can benefit from HT (Brinton, 2005). Mechanistic analyses suggest that the shorter the delay between menopause and onset of treatment, the higher chance of favorable effects of HT on the brain (Morrison et al., 2006; Brinton et al., 2015). The timing of exposure of neurons to estrogen is thus crucial, as estrogens produce positive effects if neurons are healthy at the time of exposure, while their effects may be harmful on functionally compromised cells (Brinton, 2008). In presence of Aβ-related dysregulation of mitochondrial function and calcium homeostasis, estrogen exposure may cause deleterious effects by exacerbating neuronal damage (Chen et al., 2006). Varied outcomes from clinical trials and observational studies, as well as present meta-analysis results, support the importance of initiating HT within this critical ‘window of opportunity’.

### The ‘window of opportunity’ for estrogen action in brain

In the two decades since the WHIMS raised concerns for an increased risk of dementia associated with HT, a number of studies have concluded that there may be a critical window or period in which HT exerts neuroprotection, e.g., during perimenopause or early postmenopause, or more generally, when initiated at younger ages (Resnick and Maki, 2001; Resnick and Henderson, 2002; Maki, 2008; Maki and Sundermann, 2009; Henderson and Rocca, 2012). Treatment initiation in late-life could potentially heighten the risk instead (Maki, 2005; Lethaby et al., 2008; Barrett-Connor and Laughlin, 2009; Maki and Sundermann, 2009; Marjoribanks et al., 2012; Weber et al., 2014; Conde et al., 2021; Stute et al., 2021). This theory was born out of mostly qualitative interpretation of available data, constrained by a lack of stratified estimates concerning HT formulation and timing of initiation.

While placebo-controlled RCTs remain the gold-standard in drug testing, the WHIMS was conducted in late postmenopausal women, without severe or known menopausal symptoms, and who had likely aged passed the critical window for efficacy of HT to impact estrogenic action in brain. In the present meta-analysis, pooled estimates from the two intervention trials (Shumaker et al., 2003, 2004) and consecutive post-stopping phases (Manson et al., 2013; Espeland et al., 2015) indicate a 38% increase in dementia risk with HT among postmenopausal women ages 65 and older, which was driven by the oCEEs plus MPA combination. Unopposed treatment with oCEEs initiated in older age did not significantly elevate risk. Therefore, as observed by others (Farquhar et al., 2005, 2009; Marjoribanks et al., 2012, 2017), RCTs do not support the view that HT initiated in older age protects against dementia. It is worth noting that the absolute risk was, however, relatively low: incidence rates for probable dementia in the EPT trial were 45 vs. 22 per 10,000 person-years for oCEE plus MPA vs. placebo, and 37 vs. 25 per 10,000 person-years for oCEE vs. placebo, respectively (Shumaker et al., 2003, 2004). Overall, WHIMS results may or may not generalize to midlife women, and may or may not generalize to AD either, which was not reported as a separate study endpoint.

Given the nearly 20-year gap between the average age at menopause and the average age of AD onset, RCTs using dementia incidence as the primary outcome are poorly suited to test for effects of HT started during the symptomatic menopausal phase. Prospective studies and retrospective examination of public health data and electronic medical records of large population cohorts may provide greater predictive validity for long-term AD risk, as these reports typically assess HT introduced in midlife women treated for menopausal symptoms and administered over a longer period of time. Our meta-analysis of observational studies indicates a 22% reduced risk of AD [RR = 0.778, 95% C.I. 0.639–0.948, *p* = 0.013] and 19% reduced risk of all-cause dementia [RR = 0.811, 95% C.I. 0.70–0.94, *p* = 0.007] with overall HT use.

Additionally, the current meta-analysis provides a statistical examination of the relationships between HT, its specific formulations, and timing relative to AD and dementia risk. Herein, stratification based on these parameters lends support to the window of opportunity hypothesis showing that midlife HT use was overall protective, with a significant 32% risk reduction with ET and a marginal 23% risk reduction with EPT, whereas late-life HT use was associated with an increased, albeit borderline risk of dementia. More studies with larger sample sizes are needed to confirm these results and test whether these effects vary with longer duration of use.

### Comparison with previous meta-analyses

The present meta-analysis includes the largest sample to date, including reports up to the year 2023, and takes into account known sources of heterogeneity such as type of study (RCT vs. observational study), timing of use (midlife vs. late-life), as well as type of therapy (ET vs. EPT) from the outset. Stratification based on these parameters expands on previous meta-analyses by enabling us to integrate data across various types of studies. Previous meta-analyses did not capture these effects, for several reasons. Over half were completed almost a decade ago, when limited prospective studies, and in some instances no clinical trials had been conducted (Yaffe et al., 1998; Hogervorst et al., 2000; LeBlanc et al., 2001; Farquhar et al., 2005, 2009; Marjoribanks et al., 2012; O’Brien et al., 2014). Other meta-analyses capitalized on the WHIMS RCTs excluding observational studies (Farquhar et al., 2005, 2009; Marjoribanks et al., 2012, 2017), while others included only observational studies based on concerns around enrollment bias in the WHIMS (Song Y. J. et al., 2020). Others still reported a combination of RCTs and various observational studies but did not evaluate timing of initiation (Wu et al., 2020; Zhang et al., 2021). Due to differing selection criteria, the three most recent and largest meta-analyses yielded contrasting results (Song Y. J. et al., 2020; Wu et al., 2020; Zhang et al., 2021). The first study, which consolidated data from 16 observational studies but excluded the WHIMS, showed a 33% reduced risk of AD with overall HT use (Song Y. J. et al., 2020). The second meta-analysis, combining estimates from the WHIMS and 19 observational studies, indicated an 8% increase in AD risk with HT use (Wu et al., 2020). The association was stronger with EPT while ET presented a lower but significant risk (Wu et al., 2020). Nonetheless, an inverse relationship between duration of HT use and AD risk was noted, with risk increasing in the first 5 years of use and decreasing afterwards (Wu et al., 2020). The last meta-analysis evaluated HT effects on various health outcomes including AD (Zhang et al., 2021). By pooling estimates from prior meta-analyses and 12 observational studies, EPT was associated with a 42% increase in risk of AD while ET was associated with 24% risk reduction [RR = 0.76, 95% C.I. 0.60–0.96] (Zhang et al., 2021).

Collectively, the most robust finding in our study was that ET initiated in midlife, likely in response to menopausal symptoms, had moderate positive effects on AD risk reduction. These data provide statistical integration of prior evidence of positive effects of ET (Yaffe et al., 1998; Hogervorst et al., 2000; LeBlanc et al., 2001; Song Y. J. et al., 2020; Zhang et al., 2021) or of midlife use (Yaffe et al., 1998; Hogervorst et al., 2000; LeBlanc et al., 2001; Wu et al., 2020). Additionally, a longer duration of use was associated with protective effects, in agreement with some previous reports (Hogervorst et al., 2000; Wu et al., 2020). This is clinically relevant as cohort studies have reported an almost doubled long-term risk of dementia with surgical menopause (Rocca et al., 2007, 2014; Phung et al., 2010; Bove et al., 2014). Dementia risk is generally highest following bilateral oophorectomy, intermediate with unilateral oophorectomy, and lowest but significant following hysterectomy without oophorectomy (Yaffe et al., 1998; Hogervorst et al., 2000; LeBlanc et al., 2001; Rocca et al., 2007; Phung et al., 2010; Bove et al., 2014; Gilsanz et al., 2019). However, oophorectomy after the onset of menopause was not associated with increased AD risk (Imtiaz et al., 2014), and HT initiated within a 5-year post-operatory window and continued for at least 10 years post-surgery was associated with less global cognitive decline in surgical menopausal patients (Bove et al., 2014). These observations suggest that, while the risk of dementia increases with surgical menopause, it may be reduced when estrogen therapy is initiated within the ‘critical window’ and continued for an extended period of time.

Additionally, we observed an association between overall EPT use and an elevated dementia risk, consistent with earlier reports (Wu et al., 2020; Zhang et al., 2021). This adverse outcome may be attributed to the antagonizing effect of progesterone on ERs, which may alter and possibly counteract estrogen’s neuroprotective properties (Nilsen and Brinton, 2003). The type of progestogen within a HT formulation can also impact treatment outcomes. Various progestogens have been utilized in combined HT, including progesterone and synthetic progestins such as MPA, dydrogesterone, norethindrone/norethisterone, norethisterone acetate, and levonorgestrel. Distinct progestogens have shown varying effects on HT-related risks. For instance, a relatively low risk of breast cancer and venous thromboembolism (VTE) among dydrogesterone users, and a higher risk of both condition among MPA users (Kim and Brinton, 2020). In terms of AD risk, most participants in published studies were using oral progestins, especially MPA, which may have contributed to the increased AD risk (Kim and Brinton, 2020). However, previous studies were unable to test for EPT effects in relationship to timing of use. Our stratified analysis indicates differential effects of EPT on dementia risk based on timing, in keeping with the healthy cell bias hypothesis. While late-life EPT posed an increased risk, the estimates were only of borderline significance. Therefore, the evidence is not strong enough to conclude that late-life EPT use is causative of increased dementia risk. Additionally, EPT usage during midlife leaned toward a reduction in risk, which is encouraging since EPT has generally been deemed riskier than ET (Manson et al., 2006; Lobo, 2017). Given that most women reach menopause with an intact uterus, our findings emphasize the need for continued research. A broader range of studies, especially RCTs, is needed to more conclusively evaluate the impact of EPT on dementia risk, as well as the effects of specific progestogens and administration routes.

### A critical review of research on HT for dementia prevention

Conducting studies on the impact of HT on AD and dementia incidence presents significant challenges due to inherent limitations in both RCTs and observational studies. First, RCTs did not evaluate the impact of HT initiated during the menopausal transition – the critical period for which estrogen therapy was developed. No clinical trials have included perimenopausal women to determine the benefits of HT on preventing or delaying dementia, which represents a missed opportunity. Moreover, by design, participants were uniformly treated with a single HT dose, duration of therapy, and type of estrogen and/or progestogen. There are no RCTs that examined effects of other formulations, such as transdermal estrogen, oral 17β-estradiol, or micronized progesterone, on AD or dementia risk. Although few observational studies provide estimates by administration route (Kim et al., 2021), reported a reduced risk of AD and dementia with use of oral and transdermal estrogen. Further, the EPT used in WHIMS differed from preparations used in many of the observational studies. It is possible, but still largely conjectural, that low dose transdermal estradiol with cyclic micronized progesterone, rather than a continuous oral estrogen–progestogen preparation, might lead to better outcomes than thus far achieved. A recent case–control study indicated a higher risk of dementia with EPT via both continuous and cyclic administration (Pourhadi et al., 2023), which awaits replication.

Lack of control for potential confounders such as prior history of HT use and underlying neurological status further limit interpretation of RCT results. The emergence of dementia symptoms in the presence of AD pathology may depend in part on the health of the cerebral vasculature and on concomitant pathologies. It is possible that the older postmenopausal women enrolled in the WHIMS may have harbored pre-existing cerebrovascular or neurodegenerative conditions, which may have been exacerbated by initiating therapy at that stage, possibly accelerating dementia onset in turn (Resnick and Maki, 2001; Resnick and Henderson, 2002; Maki, 2008; Maki and Sundermann, 2009; Henderson and Rocca, 2012). Studies incorporating brain imaging or other AD biomarkers suggest that a patient’s underlying neurological health may indeed be consequential. The WHIMS reported that HT was associated with greater brain atrophy in postmenopausal women compared to placebo (Resnick et al., 2009), with more severe reductions in hippocampal volume in those with lower cognitive function prior to initiating treatment (Resnick et al., 2009). Presence of multi-infarcts may also have played a role, as MPA has been associated with an increased risk of vascular disease (Irwin et al., 2011). While excluding women with stroke in post-hoc analysis did not alter the association between HT and dementia incidence (Shumaker et al., 2003), it remains unknown whether microvascular events or other insults such as lacunar infarcts and high-grade deep white matter lesions could have been involved in the increased dementia risk. Younger women are less likely to exhibit significant AD neuropathology or cerebrovascular insults, which may support HT safety and efficacy when instituted in midlife. To this point, in the WHIMS of Younger Women (WHIMS-Y), there was neither a beneficial nor a harmful effect of HT on long-term cognitive function in postmenopausal women receiving oCEEs at earlier ages of 50–55 years (Espeland et al., 2013). The ELITE-cog and KEEPS studies have also reported neutral effects of HT on cognition among recently postmenopausal women (Shumaker et al., 2003, 2004; Gleason et al., 2015; Hodis et al., 2015). Further, HT reduced the progression of subclinical atherosclerosis when therapy was initiated soon after menopause (Hodis et al., 2016), which has been linked to a 30% reduced number of heart attacks and cardiac deaths (Salpeter et al., 2009).

Observational studies are also subject to limitations. First, case–control and cross-sectional studies requiring AD patients and/or an informant to provide details about their past history of HT use are impacted by concerns around recall bias. Recall bias is less likely in studies where information on hormone use was collected before the onset of dementia, such as in prospective studies. Herein, we did not find clear differences in risk estimates between case–control and cohort studies, although the former group carried a slightly larger effect size with 20% reduced risk of AD or dementia vs. 16% for cohort studies. Confounding by the healthy user effect is another concern in observational research, and so is “confounding by indication,” e.g., women using HT during menopause may experience more severe menopausal symptoms, such as vasomotor symptoms, sleep disturbances, depressive symptoms, and cognitive changes, than those who do not take hormones. All these symptoms have been linked to a higher risk for dementia in turn (Brinton et al., 2015; Livingston et al., 2020). On the other hand, women who receive HT tend to be healthier, have higher education, and be more socioeconomically advantaged than never-users, a phenomenon known as the ‘healthy user-bias’ (Matthews et al., 1996). HT usage may therefore be associated with a healthier lifestyle which in turn might be preserving cognitive function independent of, or in combination with HT. Further, women with access to HT are more likely to have better-quality health care overall, which can decrease risk for AD or dementia independent of HT use (Matthews et al., 1996). Nonetheless, variables that are reflective of these effects, such as education and socioeconomical status, were accounted for in several studies. Since RRs that control for known potential confounders are likely more accurate in estimating risk compared to unadjusted RRs, this meta-analysis used adjusted risk estimates whenever available, which was the case for the vast majority of studies. Lastly, the majority of observational studies, particularly those prior to 2010, compared the broad categories of HT users vs. non-users, and did not test for effects of HT type, dosage or duration.

While we observed substantial heterogeneity in effect size across the studies, our meta-regression analysis found that heterogeneity was not significantly related to factors such as study design, duration of use, sample size, publication year, or estimate type. The primary determinants of the observed heterogeneity in the association between HT and AD risk appeared to be two specific variables: the age at usage, with midlife use showing a protective effect, and the type of HT used, where ET exhibited a more pronounced effect compared to EPT. Therefore, we categorized studies based on these variables and re-conducted the meta-analysis. Stratification by midlife use and by ET status yielded an increased effect size, supporting the long-standing hypothesis that beginning ET in midlife, or close to menopause onset, may be protective benefits against AD. Nonetheless, heterogeneity persisted, underscoring the potential limitations present within the existing literature and emphasizing the need for caution in interpreting the findings. We recommend that future research endeavors adopt more standardized methodologies, ensuring uniform exposure and outcome criteria, to enhance consistency and comparability across studies. Given our findings, it seems important for future epidemiological studies and RCTs to prioritize research on the effects of midlife ET use when examining its association with AD risk.

### HT effects on cognition and AD biomarkers

Additional evidence in support of HT use for AD risk reduction stems from studies investigating cognition and biomarkers of AD risk in postmenopausal women. While the results of these studies have been conflicting, the majority show that estrogen may facilitate maintenance of some aspects of cognition (Kampen and Sherwin, 1994; Robinson et al., 1994; Kimura, 1995; Szklo et al., 1996; Resnick et al., 1998; Hogervorst et al., 2000), particularly verbal memory (LeBlanc et al., 2001; Maki, 2005; Maki and Sundermann, 2009), when initiated in early postmenopause or prior. There is also indication of positive, yet mild effects of HT on learning and processing speed (Maki and Sundermann, 2009). Effects vary, however, with HT type, timing, and overall neurocognitive health prior to menopause, with more consistent benefits for ET (LeBlanc et al., 2001; Maki, 2005; Maki and Sundermann, 2009).

While neuroimaging studies of HT are scarce, some reports suggest a positive role of estrogen therapy on CMRglc (Eberling et al., 2000; Rasgon et al., 2005, 2014; Silverman et al., 2011; Rahman et al., 2020), cerebral blood flow (Maki and Resnick, 2000; Slopien et al., 2003), Aβ deposition (Kantarci et al., 2016a; Rahman et al., 2020) and tau pathology (Wisch et al., 2021). Additionally, MRI studies provided conflicting results of larger brain and regional volume in HT users vs. non-users or placebo (Eberling et al., 2003; Erickson et al., 2005, 2010; Boccardi et al., 2006; Lord et al., 2008; Albert et al., 2017; Rahman et al., 2020; Boyle et al., 2021; Schelbaum et al., 2021; Saleh et al., 2023), and vice versa (Greenberg et al., 2006; Low et al., 2006; Resnick et al., 2009; Coker et al., 2014; Ryan et al., 2014; Zhang et al., 2016), as well as lack of differences (Ryan et al., 2014; Braden et al., 2017). However, studies of postmenopausal women in their 60s or younger yielded more generally positive results, whereas negative reports included mostly women of advanced age (Resnick et al., 2009; Zhang et al., 2016), sometimes with scanning conducted several years after HT ended (Ryan et al., 2014; Braden et al., 2017). There are also some reports of increased white matter hyperintensities with HT use (Kantarci et al., 2016b, 2018) although results are again mixed (Coker et al., 2014; Zhang et al., 2016; Jayachandran et al., 2020; Kling et al., 2020), suggesting that HT effects are either small or moderated by confounders, such as age and underlying cardiovascular health. Notably, a recent prospective, interventional study assessed plasma biomarkers of Aβ pathology, tauopathy, and neurodegeneration in 224 cognitively healthy recently postmenopausal women, drug-naïve for HT at inclusion. Of these, 193 elected to start HT as part of the study and were compared to 31 untreated controls. Over a 6-month period, HT use was associated with lower rates of AD biomarker change as reflected in a smaller reduction in Aβ_42_/p-tau_231_ ratios, which was more among APOE4 carriers (Depypere et al., 2022), suggesting possible gene–environment effects.

### Future directions and considerations for clinical practice

Active debate remains on whether HT has value for the clinical practice of AD risk reduction. Most interpretations thus far have relied on data from RCTs, which besides being extremely scarce, were late intervention studies of older postmenopausal women without active menopausal symptoms. These women are not representative of those requiring HT in real world clinical settings. Natural history studies and retrospective analysis of electronic records of younger women, which may have better captured the critical window for HT action, indicate that HT may indeed play a role in AD prevention for perimenopausal and recently postmenopausal women, especially surgical menopausal patients.

The GRADE (Grading of Recommendations, Assessment, Development, and Evaluations) framework is commonly used to summarize the quality of evidence for making clinical practice recommendations (Guyatt et al., 2008). In GRADE, only RCTs are initially rated as high-quality evidence, while observational studies are considered low-quality evidence by default. As only one RCT investigated HT use relative to dementia incidence, and the remaining evidence base is exclusively observational, the current literature provides an overall low-level evidence for mild to moderate protective effects of HT in midlife and a possible elevation in risk with late-life use. Consequently, these findings do not warrant clinical application, but underscore the urgent need for further research in this domain. Given the need for large sample sizes and lengthy follow-up, it is difficult to envision primary prevention trials for AD that begin in midlife. Well-designed cohort studies corroborated by a firmer understanding of basic mechanisms of estrogen action in brain, and long-term clinical trials using surrogate biomarkers of AD pathology and related functional changes represent an attractive opportunity to answer remaining questions.

Findings of protective effects of HT in women with surgical menopause are of particular relevance to clinical practice given that estrogen therapy (with endometrial protection if the uterus is preserved) may reduce risks of cognitive and affective disorders for women with premature or early menopause, if administered early and taken until the average age of menopause (North American Menopause Society, 2022). Present findings of reduced AD risk with ET following hysterectomy/oophorectomy align with this position, and corroborate current emphasis on mitigating modifiable AD risk factors as a preventative strategy. The *Lancet* Commission identified twelve potentially modifiable dementia risks, including medical risks such as hypertension, obesity, depression, diabetes, which collectively account for 40% of dementia cases worldwide (Livingston et al., 2020). It was estimated that delaying the onset of AD by just 1 year could reduce AD cases in the over-60s by 11% (Norton et al., 2014), while a five-year delay could lead to a 41% reduction in prevalence by 2050 (Andrieu et al., 2015). Within this context, estrogen insufficiency represents a modifiable, female-specific risk factor for AD, warranting particular consideration for women who undergo hysterectomy. Although the relative risk associated with non-use of HT is modest, the potential societal benefits may be substantial given the size of the affected population.

Estrogen action in brain is dependent on multiple factors, including chronological age, stage of reproductive aging, duration of hypogonadism, and presence of symptoms, as well as the formulation of HT, route of administration, a patient’s medical history and the health status of the brain (Manson et al., 2006; Lobo, 2017; Arnold et al., 2020). This calls for an individualized approach to HT usage to increase safety and predictive efficacy for AD prevention and preservation of neurological function, based on assessment of not only the patient’s age, presence of menopausal symptoms, and age at peri/menopause, but also of underlying medical, neurological and cardiovascular health risks. While the former set of evaluations are standard practice during OBGYN evaluations, the latter call for an integrative approach to menopause involving neurological specialists, among others.

Assessments of cognitive performance using screening tools such as the Mini-Mental Status Examination (MMSE), Montreal Cognitive Assessment (MoCA), and the Functional Assessment Questionnaire (FAQ), as well as standardized testing batteries, can be helpful to address cognitive concerns in patients of menopausal age. While cognitive symptoms due to menopause may not result in significant changes on these tests, they represent a suitable strategy to differentiate subjective from objective complaints, and reassure patients that their complaints may arise from transient cognitive decline associated with menopause rather than dementia. Repeated cognitive assessments may be especially valuable for women who undergo early or surgical menopause. If cognitive impairment is detected, a thorough neuropsychiatric and neuropsychological evaluation is warranted, along with laboratory workup and neuroimaging, to investigate possible underlying causes.

Additionally, more precise ways of probing the health status of the brain are warranted, especially among older women. Medical imaging examining presence of cerebrovascular and neurodegenerative insults can further guide both research and clinical practice on optimal intervention and timeline for any given individual. MRI is widely used to gauge presence of brain atrophy and ventricular enlargement, presence of white matter hyperintensities, microbleeds, and other signs of brain insults that may worsen with HT. PET imaging and blood-based biomarkers of AD pathology are not currently approved for screening of asymptomatic at-risk individuals, but their value is becoming increasingly clear. Moving forward, use of biomarkers specifically assessing neurological health and underlying AD risk before starting HT, and throughout treatment duration, may provide important information for individualized treatment.

More effort is also needed in the area of drug discovery. For instance, previous work has shown that orally administered estrogen leads to a hepatic concentration of E2 that is 4 to 5 fold higher than serum levels of E2 due to the first-pass effect, e.g., the absorption and transport of oral drugs from the gut to the liver (Menon and Vongpatanasin, 2006). This supra-physiological dose of E2 may lead to hepatic elevation of metabolites such as inflammatory C-reactive protein, serum Amyloid A, prothrombin fragments, and atherosclerotic plaque-disrupting enzymes (Menon and Vongpatanasin, 2006). These findings help to explain why oCEEs have been associated with increased risks for VTE and ischemic stroke, and cerebrovascular disease in turn (Taylor and Manson, 2011). In contrast, transdermal E2 bypasses the gut and liver entirely, providing a likely safer option (Taylor and Manson, 2011). It is plausible that transdermal E2 may provide the same cognitive benefits as oral estrogen without elevating cardiovascular or neurological risk, though this remains to be confirmed.

Finally, both oral and transdermal routes carry other potential risks, such as the development of reproductive cancers. While the risk of endometrial cancer is reduced by combining estrogen with progestogen, HT has been linked with a small but significant risk of breast and ovarian cancer with longer duration of use (Lobo, 2017; North American Menopause Society, 2022). HT is not currently recommended for women with a previous history of cancer, among other contraindications. A major goal in the field is to develop compounds that exert the neuroprotective effects of estradiol while circumventing the potential risks associated with HT. To this aim, a class of compounds known as neuro-SERMs (neurological selective estrogen receptor modifiers) have been developed, which can selectively traverse the blood–brain barrier and exert neuroprotection without acting on peripheral reproductive tissues or causing unwanted estrogenic side-effects (Schneider et al., 2019). A Phase IIb randomized, placebo-controlled clinical trial testing the efficacy of PhytoSERM, a selective ERβ modulator comprised of three phytoestrogens: genistein, daidzein, and S-equol (Zhao et al., 2009), for support of bioenergetic and cognitive function in midlife women at risk for AD is underway.

## Conclusion

Results of the present meta-analysis suggest that estrogen therapy initiated during the critical window of the menopause transition may support neurological function and reduce the risk of future AD among eligible women. We recognize and emphasize the inherent limitations of relying largely on observational data and that stronger, randomized controlled trial evidence is needed to fortify these conclusions. Overall, these findings provide new insights on the association between HT use and AD incidence, and support renewed research interest in evaluating HT for the purpose of AD and dementia risk reduction.

## Data availability statement

The original contributions presented in the study are included in the article/Supplementary material, further inquiries can be directed to the corresponding author.

## Author contributions

MN: Writing – original draft, Data curation, Investigation. SJ: Writing – original draft, Data curation, Investigation. CA: Writing – original draft, Formal analysis, Methodology. CC: Writing – review & editing. CZ: Writing – review & editing. CB: Writing – review & editing. MB: Writing – review & editing. SP: Writing – review & editing. SL-Z: Writing – review & editing. YH: Writing – review & editing. SW: Writing – review & editing. PC: Writing – review & editing. MF: Writing – review & editing. RB: Writing – review & editing. LM: Writing – original draft, Writing – review & editing, Conceptualization, Funding acquisition, Project administration, Resources, Supervision.
