# Supplementary material for: Systematic review and meta-analysis of the effects of menopause hormone therapy on risk of Alzheimer’s disease and dementia
Source: Front Aging Neurosci. 2023 Oct 23;15:1260427. doi: 10.3389/fnagi.2023.1260427 (PMC10625913; doi:10.3389/fnagi.2023.1260427)
Supplement: Supplementary file 1 [file Data_Sheet_1.DOCX]

## Supplementary Table 1. Meta-regression analysis

|  | **Estimate** | **Lower 95% CI** | **Upper 95% CI** | **P** |
| --- | --- | --- | --- | --- |
| Intercept | 0.729 | 0.465 | 0.993 | <0.001 |
| Dementia | -0.003 | -0.028 | 0.021 | 0.778 |
| Estrogen-only | -0.075 | -0.114 | -0.036 | <0.001 |
| Estrogen-progesterone | -0.044 | -0.082 | -0.007 | 0.021 |
| Midlife | -0.097 | -0.132 | -0.063 | <0.001 |
| Late life | -0.047 | -0.096 | 0.002 | 0.06 |
| Long duration of use | 0.012 | -0.015 | 0.039 | 0.374 |
| Short duration of use | 0.004 | -0.020 | 0.027 | 0.768 |
| Case-control study design | 0.105 | -0.110 | 0.320 | 0.34 |
| Cross-sectional study design | 0.080 | -0.427 | 0.588 | 0.756 |
| Sample size < 500 | 0.031 | -0.160 | 0.223 | 0.748 |
| Time period: Before 1995 | -0.093 | -0.294 | 0.107 | 0.362 |
| Time period: 1995-2010 | 0.029 | -0.242 | 0.301 | 0.832 |
| Effect estimate: OR | 0.065 | -0.217 | 0.348 | 0.651 |
| Effect estimate: HR | 0.231 | -0.016 | 0.479 | 0.067 |

## Supplementary Figure 1. Meta-analysis of the unrestricted observational dataset including separate arms for estrogen-only and estrogen-progesterone therapy

##
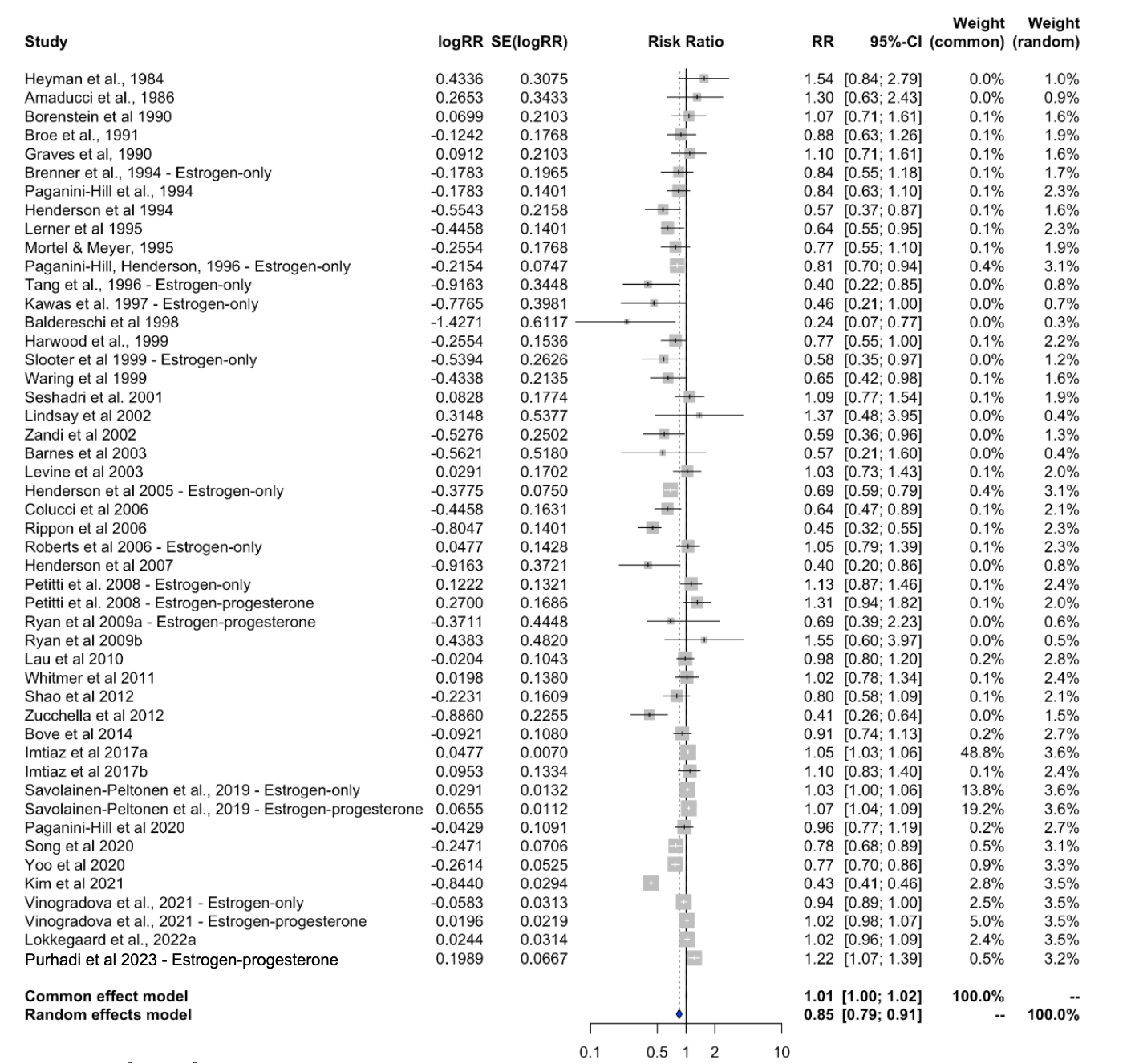


Meta-analysis of observational studies examining the risk of developing AD or dementia from the unrestricted dataset, including specific arms. Forest plots display individual and pooled estimates of the association between use of HT and risk of AD or dementia expressed as relative risk (RR) and 95% confidence intervals (C.I.). Studies are ordered by year of publication.

## Supplementary Figure 2. Examination of publication bias


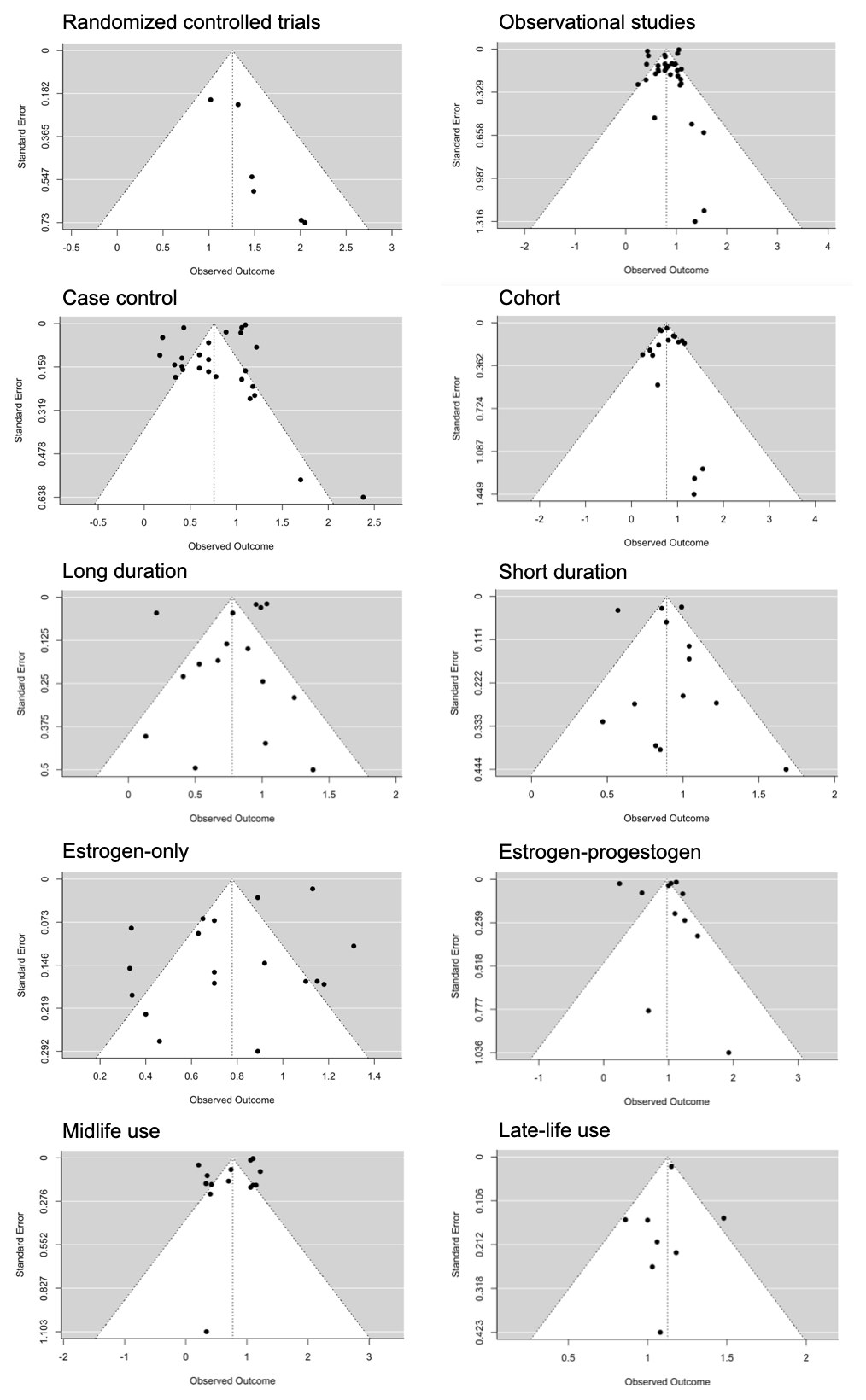


Funnel plots of standard error by outcome assessing publication bias.
